# Association Between β‐Adrenoreceptor Agonists and Antagonists and Parkinson's Disease: Systematic Review and Meta‐Analysis

**DOI:** 10.1002/pds.70140

**Published:** 2025-04-09

**Authors:** Agnieszka Szmigiel, Miguel Monteiro da Rocha, Kate Browne, Daniel Morales, David Benee Olsen, Charlotte Warren‐Gash, Ian Douglas, Krishnan Bhaskaran, Helena Carreira

**Affiliations:** ^1^ Pharmacovigilance Office European Medicines Agency Amsterdam the Netherlands; ^2^ Faculty of Epidemiology and Population Health London School of Hygiene & Tropical Medicine London UK; ^3^ North Region Health Administration National Health Service Lisbon Portugal; ^4^ Medical and Health Information Service European Medicines Agency Amsterdam the Netherlands; ^5^ Real World Evidence European Medicines Agency Amsterdam the Netherlands; ^6^ Department of Pharmacovigilance Norwegian Medical Products Agency Oslo Norway

**Keywords:** adrenergic beta‐agonists, adrenergic beta‐antagonists, Parkinson's disease, systematic review

## Abstract

**Background:**

β‐agonists and β‐antagonists are among the most prescribed drugs worldwide. In 2018, studies suggesting a harmful association between propranolol and Parkinson's disease (PD) prompted a signal procedure by the European Medicines Agency's safety committee, which concluded with no update of product information. Several studies have been published since then. We aimed to systematically review, critically appraise, and meta‐analyse all studies on the association between the use of β‐antagonists (including propranolol) and β‐agonists, and the risk of PD.

**Methods:**

We searched Embase and Medline up to December 2024 for observational and intervention studies that reported relative risk estimates of the association between use of these medicines and PD. Two reviewers screened the records, extracted the data, and assessed the risk of bias. The restricted maximum likelihood method was used to compute pooled effect estimates and 95% confidence intervals (CIs).

**Results:**

Twenty‐two studies were eligible. Overall, 20 had a high risk of bias in at least one domain. Twelve studies had medium to high risk of outcome misclassification. Of the 14 studies concerning β‐antagonists, eleven had an unclear or high risk of protopathic bias, as propranolol is indicated for the treatment of essential tremor. Control for confounding by socio‐economic status, area of residence (urban/rural), and smoking (a protective factor against PD) was deficient or lacking in 9/22, 15/22, and 12/22 studies, respectively. Lag times were applied in 9/22 studies. In meta‐analysis, the summary relative risk (RR) of PD was 1.41 (95% CI: 1.18–1.68) for the class of β‐antagonists (12 studies) and 0.93 (0.84–1.03) for β2‐agonists (11 studies). Among specific β‐antagonists, the summary RR of PD was 2.36 (1.66–3.36) for propranolol (7 studies), 0.84 (0.80–0.88) for carvedilol (3 studies) and 1.02 (0.87–1.18) for metoprolol (4 studies). For specific β2‐agonists, summary RR was 0.88 (0.77–1.01) for salbutamol (7 studies), 0.91 (0.88–0.95) for short‐acting β2‐agonists (6 studies), and 0.85 (0.76–0.96) for long‐acting β2 agonists (5 studies). Restricting to subgroups based on quality criteria resulted in weaker or non‐statistically significant associations.

**Conclusion:**

The quality and quantity of the available evidence do not support a causal association between use of β‐adrenoreceptor modulators and PD. Significant associations are most likely explained by protopathic bias and confounding.

**Summary**

- This systematic review included 22 observational studies published between 2007 and 2024 on the association between β‐adrenergic drugs and Parkinson's disease risk.
- Most studies were subject to important biases that may explain the associations observed in meta‐analyses.
- In meta‐analyses, β‐antagonist users had a 41% increased risk of PD compared to never users, and β2‐agonist users had a 7% non‐significant reduced risk of PD. There was a possible dose–response relationship between the short‐acting versus long‐acting agents, though publication bias was also detected.
- The available epidemiologic data are not sufficiently consistent to infer a causal relationship between the use of β‐adrenergic drugs and the risk of PD.

## 1 Introduction

Parkinson's disease (PD) is a complex neurodegenerative condition that often presents with progressive bradykinesia, rest tremor, rigidity, and lack of postural reflexes [1]. In 2016, over 6.1 million people were estimated to be living with the disease globally [2]. The burden of PD in high‐income settings increased in the last decades, particularly among men [3], for unknown reasons [2].

The pathophysiology of PD includes an abnormal accumulation of α‐synuclein protein in the brain. In 2017, Mittal et al. [4] showed that the β2‐adrenoreceptor (β2‐AR) regulates the transcription of the human α‐synuclein gene and postulated that β2‐AR ligands may modulate the risk of PD. The authors then conducted a cohort study using data from a Norwegian prescription database and reported that the use of propranolol (a non‐selective β‐blocker) was associated with a 2‐fold increased risk of PD, while salbutamol (a β2‐agonist) had a dose‐dependent protective association [< 60 Defined Daily Dose (DDD): RR 0.96 (95% CI 0.76–1.23); 60–180 DDD: 0.60 (0.40–0.91); > 180 DDD: 0.45 (0.31–0.67)] [4]. Other studies, some of which directly aimed to replicate the associations shown by Mittal et al. [4], showed conflicting results [5, 6]. These results generated interest, as drugs acting on the βAR have a broad use in medicine as well‐established treatments for common chronic conditions such as hypertension, asthma, chronic obstructive pulmonary disease (COPD) and angina [7]. The Mittal et al. study prompted a safety signal procedure at the European Medicines Agency (EMA) in June 2018, in line with the EU Guideline on Good Pharmacovigilance Practices, when new data suggests a potentially causal association or a new aspect of a known association [8]. At the time, the Pharmacovigilance Risk Assessment Committee (PRAC) concluded that there was not enough evidence to update product information, as the available studies often did not adjust for important confounders such as smoking (associated with decreased risk of PD [9]) or account for a time lag between exposure to β2‐AR drugs and incidence of PD. This is important because some β2‐AR are indicated for the treatment of essential tremor [10], which may be early signs of PD [11]. Studies were also heterogeneous in the definition of the exposure (whole class versus specific drugs only), type of population studied, and duration of follow‐up, all of which may affect the results. Several studies have been published since then.

In this study, we aimed to systematically review, critically appraise, and meta‐analyse the available evidence on the association between exposure to β2‐AR drugs (β‐antagonists, β‐agonists) and the risk of PD to date. We considered studies providing data for the broad classes of β‐antagonists and β‐agonists, as well as individual drugs within these classes, and comprehensively discussed the potential for bias in the available evidence.

## 2 Methods

This systematic review is reported in accordance with the Preferred Reporting Items for Systematic Reviews and Meta‐Analyses (PRISMA) guidelines statement [12]. The systematic review protocol was registered with the international prospective register of systematic reviews (PROSPERO, CRD42020219419).

### 2.1 Study Inclusion Criteria

We identified studies that quantified the association between the use of β‐antagonists and/or β‐agonists and the risk of developing PD following the PECOS framework [13]: (1) Population: Individuals at risk of PD; (2) exposure: use of drugs belonging to the classes of β‐antagonists [Anatomical Therapeutic Chemical Classification System (ATC) codes: C07AA (non‐selective), C07AB (selective) and C07AG (alpha and beta)] and β‐agonists [ATC codes R03AC (selective β2‐agonists) and R03AK, R03AL (adrenergic in combination) for inhalants; and R03CC (selective β2‐agonists) for systemic use]; (3) comparator: unexposed group or lowest exposure category when used as a reference; (4) outcome: PD; (5) study design: intervention, cohort, or case control; (6) reported effect estimates.

### 2.2 Literature Search

We queried Medline and Embase (both from inception up to December 2024) to identify eligible studies. The search expressions included terms for the exposure (β‐antagonists, β‐agonists and respective drugs) and outcome (PD) in the form of Medical Subject Headings (MeSH) and text words (Appendix S1). To avoid missing relevant studies, we manually screened references of the eligible studies and related reviews. There were no time, geographical, or language restrictions.

### 2.3 Data Management and Screening of References

Records from Medline and Embase were exported to Endnote X20. Duplicates were removed manually. The titles and abstracts were systematically screened by two reviewers (AS, HC) for relevance according to the inclusion criteria (listed above). The full text of potentially relevant studies was obtained and reviewed by two reviewers to determine the final eligibility for this review. Study authors were contacted for additional information to clarify relevant aspects.

### 2.4 Data Extraction

The following information was extracted using a pre‐defined data extraction form: (1) country; (2) study design; (3) study population; (4) number of people exposed and unexposed (or exposed to the lowest dose); (5) mean/median age of participants and their sex (as percentage); (6) drug class (β‐antagonists, β‐agonists), and/or individual drugs (e.g., propranolol, salbutamol); (7) effect estimates and 95% confidence intervals; (8) control of confounding by socio‐demographic factors (age, sex, socio‐economic status), urban/rural residence, smoking, others; (9) time lag between exposure and start of outcome ascertainment (to assess the possibility of reverse causality); (10) dose–response results; and (11) length of follow‐up. When both crude and adjusted measures of effect were provided, we extracted both. When a study provided results from analyses of two or more databases of electronic health records, we extracted all data but only considered the study once in the analysis.

### 2.5 Risk of Bias in Individual Studies

To evaluate the risk of bias of the included studies, we selected domains that are important in observational pharmacoepidemiology studies using routinely collected healthcare data [14, 15]. The domains were: validity of exposure and outcome ascertainment; protopathic bias; control for confounding by age, sex, socio‐economic status [16], area of residence (urban/rural), as PD is more common in rural areas [17], and smoking [9]; evaluation of dose–response relationship; generalisability (external validity); and conflict of interests. Within each domain, the studies were rated as having a high, moderate/unclear, or low risk of bias by two researchers, who also reconciled any disagreement by a consensus. Appendix S2 provides the criteria used for each category and domain.

### 2.6 Data Synthesis

Data on the studies' characteristics and results were summarised descriptively in tables and text. The results were presented grouping the studies by overall drug classes (β‐antagonists, β‐agonists), selectivity for β‐AR subtypes (selective versus non‐selective), effect duration for β2‐agonists short‐acting (SABA), long‐acting (LABA), ultra‐LABA, and individual drugs in line with their respective categories. Studies that provided results for more than one of these categories (e.g., all β2‐agonists as well as salbutamol) were included in the analyses of relevant categories.

Meta‐analysis was used to quantitatively summarise the associations between each drug and drug class and the risk of PD as reported by the relative risk, hazard ratio, or odds ratio in the original studies. In all analyses, the restricted maximum likelihood method was used to compute summary RR estimates with 95% confidence intervals (CI). This method produces unbiased estimates of the between‐study variance [18]. Heterogeneity of effects was assessed and quantified using the I^2^ statistic [19]. In the main analysis, we included the results of the studies that compared the risk of PD between those (ever) exposed to the β‐AR drug class or individual drug and those never exposed (or exposed to the lowest category); this was the most frequently reported comparison in the original studies. In sensitivity analyses, we repeated the main analysis for the drug classes, type of β‐agonists (short‐ and long‐acting), and the most studied drugs in each class (i.e., propranolol and salbutamol), using estimates for the longest duration or highest dose of exposure (e.g., use of β‐blocker for > 6 years versus never use), if available. To explore sources of heterogeneity, we ran several sub‐group analyses: restricting to studies at low risk of protopathic bias (β‐antagonists only); studies that provided estimates adjusted for potential confounders, including SES, area of residence (urban/rural), and smoking (a protective factor); and studies that employed a time lag before outcome ascertainment (to avoid reverse causality). Since important clinical and statistical heterogeneity was observed across studies, random‐effects models were chosen to incorporate the between‐study variance within the analysis. Small study bias was assessed through visual inspection of funnel plots and Egger's regression asymmetry test [20, 21]. Stata version 18 was used for analysis.

## 3 Results

**FIGURE 1:**
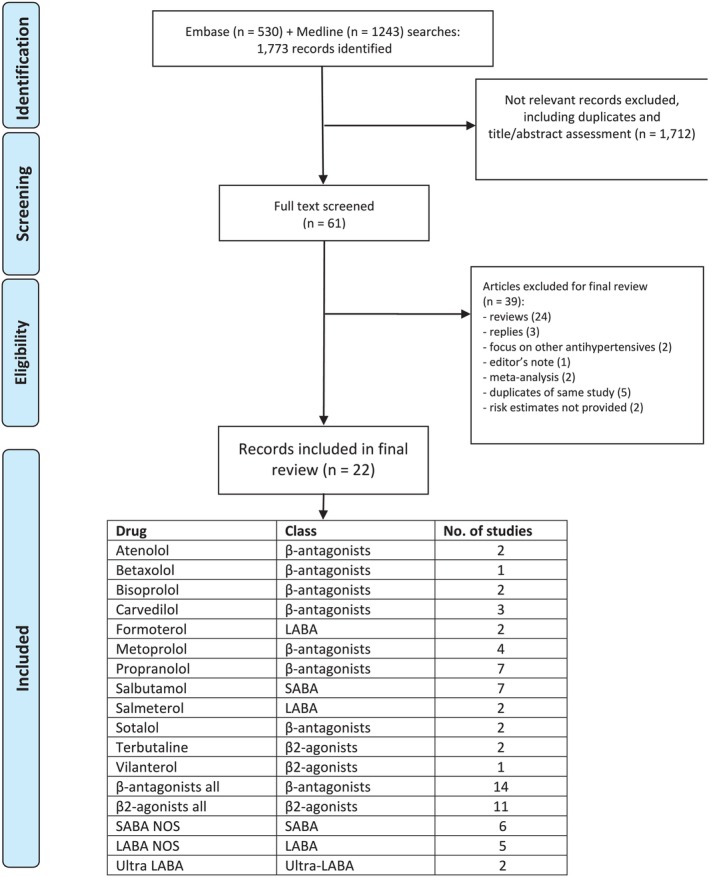
Systematic review flowchart. SABA = short‐acting beta‐agonist; LABA = long‐acting beta‐agonist; NOS = not otherwise specified.

The literature search yielded 1773 records from Embase and Medline (Figure 1). A total of 1712 articles were excluded during screening based on title and abstract, leaving 61 articles for full text assessment. Of these, 22 studies [4, 5, 6, 22, 23, 24, 25, 26, 27, 28, 29, 30, 31, 32, 33, 34, 35, 36, 37, 38, 39, 40] were eligible for the systematic review.

### 3.1 Characteristics of Included Studies

The characteristics of the included studies are shown in Table 1. All studies were observational in design and used national or commercial electronic health records databases. Fourteen were nested case–control studies, seven were cohort studies, and one was a self‐controlled cohort study. Men represented 50%–65% of PD cases in 13 studies. Sample size varied from < 1000 up to 117 million patients in a study of US cohorts. 12 studies assessed the class of β‐antagonists, while 11 studies evaluated β2‐agonists class. In studies evaluating individual β‐antagonist drugs, included propranolol (7 studies), carvedilol (3 studies), sotalol (2 studies), metoprolol (4 studies), atenolol (2 studies) and bisoprolol (2 studies) and betaxolol (1 study). Two studies [5, 25] investigated the effects of selective β1‐blockers and non‐selective drugs separately. Studies evaluating β‐agonists included individual drugs formoterol (2 studies), salbutamol (7 studies), salmeterol (2 studies), terbutaline (2 studies), and vilanterol (1 study). SABA and LABA were reported in 6 and 5 studies each, and ultra‐LABA in 2 studies. Appendix S3 provides detailed information on each study's characteristics and results.

**TABLE 1:** Summary of the main characteristics of the eligible studies (*N* = 22).

Study characteristics		Number of studies	(%)
Type of study	Longitudinal	22	(100)
Study design	Cohort study	9	(40.9)
	Nested case–control study	12	(54.5)
	Self‐controlled cohort study	1	(4.5)
Country	Canada	2	(9.1)
	Denmark	2	(9.1)
	Finland	1	(4.5)
	France	1	(4.5)
	Germany	1	(4.5)
	Israel	2	(9.1)
	Korea (South)	1	(4.5)
	Norway	2	(9.1)
	Sweden	1	(4.5)
	United Kingdom	3	(13.6)
	Taiwan	1	(4.5)
	United States	5	(22.7)
Study size	Case control studies, number of cases		
	< 1000	2	(9.1)
	1000‐5000	6	(27.3)
	8500–11 500	3	(13.6)
	> 45 000	1	(4.5)
	Cohort studies, total number of people		
	< 250 000	3	(13.6)
	1.0–4.4 million	5	(22.7)
	4.5–5.2 million	1	(4.5)
	117 million	1	(4.5)
Data source	Databases of electronic health records	22	(100)
Type of database	General population^a^	15	(68.2)
	Prescription^b^	4	(18.2)
	Commercial^c^	3	(13.6)
Type of population	Broad group of patients	18	(81.8)
	Patients with COPD	1	(4.5)
	Patients with COPD/asthma	2	(9.1)
	Patients with asthma, COPD, and/or bronchiectasis	1	(4.5)
% of men in PD cases	< 50%	2	(9.1)
	50%–65%	13	(59.1)
	Not provided	7	(31.8)
Mean age of PD	> 40 years	1	(4.5)
	53–75 years	7	(31.8)
	75–80 years	5	(22.7)
	Not provided	9	(40.9)
Exposures evaluated	Any β‐antagonist, incl.	14	(63.6)
	β‐antagonists, all^d^	12	(54.5)
	Atenolol	2	(9.1)
	Bisoprolol	2	(9.1)
	Betaxolol	1	(4.5)
	Carvedilol	3	(13.6)
	Metoprolol	4	(18.2)
	Propranolol	7	(31.8)
	Sotalol	2	(9.1)
	Any β2‐agonists, incl.	11	(50.0)
	SABA	6	(27.3)
	LABA	5	(22.7)
	Ultra LABA	2	(9.1)
	Formoterol	2	(9.1)
	Salbutamol	7	(31.8)
	Salmeterol	2	(9.1)
	Terbutaline	2	(9.1)
	Vilanterol	1	(4.5)

Abbreviations: COPD = chronic obstructive pulmonary disease; LABA = long‐acting β2‐agonists; SABA = short‐acting β2‐agonists.

^a^ UK Clinical Practice Research Datalink [29, 38], the French Echantillon Généraliste des Bénéficiaires data base [27], the Israeli Maccabi and Clalit Health Services [5, 37], the US Medicare [6, 32], the National Registries of Danish Person, Patients and Prescriptions [23, 36], the Group Health Cooperative, Ontario's health administrative databases [22, 30], Taiwan's National Health Insurance Research Database [35], the National Health Insurance Service of Korea [34], UK Biobank [33], and the Swedish health and drug prescription records linked to the Longitudinal Integration Database for Health Insurance and Labor Market Studies [40].

^b^ British Columbia health administrative databases [28], the Norwegian Prescription Database [4, 31], Finnish Prescription Register [39].

^c^ IQVIA [26], IMB commercial databases [25] or TriNetX Analytics [24].

^d^ Ton et al., 2007 included atenolol, propranolol, nadolol, metoprolol, labetalol, and carvedilol only. These represented most prescriptions of the class.

### 3.2 Risk of Bias in the Original Studies

**FIGURE 2:**
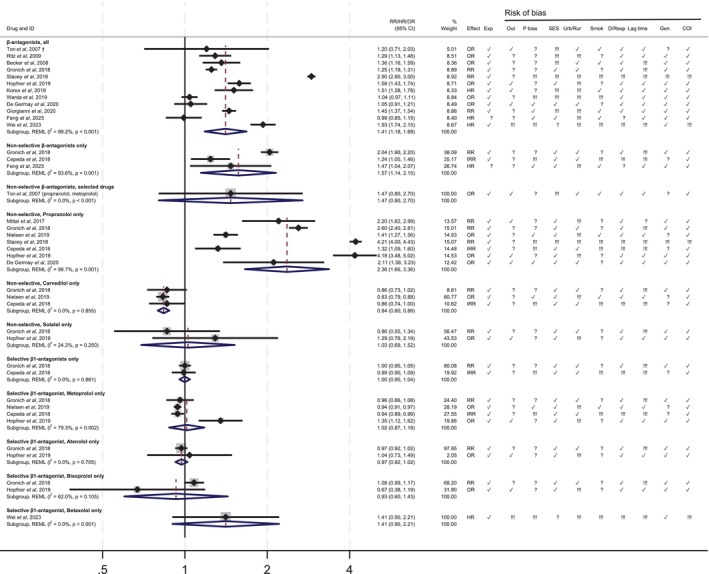
Associations between β‐antagonists and Parkinson's disease. † Ton et al., 2007 included atenolol, propranolol, nadolol, metoprolol, labetalol, and carvedilol only. Exp = misclassification of the outcome; Out = misclassification of the outcome; P bias = protopathic bias; SES= confounding by socioeconomic status (age, sex, socio‐economic status); Urb/Rur = confounding by urban/rural residence; Smok = confounding by smoking; D/Resp = dose–response relationship and/or length of exposure; Gen = generalizability; COI = conflict of interest. ✔ = low risk of bias; !!! = high risk of bias; ? = unclear risk of bias.

**FIGURE 3:**
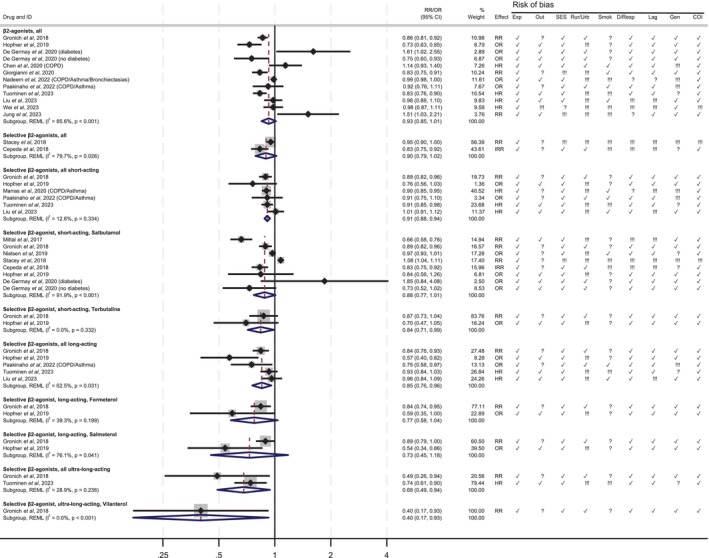
Associations between β‐agonists and Parkinson's disease. Exp = misclassification of the outcome; Out = misclassification of the outcome; C‐SES= confounding by socioeconomic status (age, sex, socio‐economic status); C‐U/*R* = confounding by urban/rural residence; C‐smoke = confounding by smoking; DR = dose–response relationship and/or length of exposure; G = generalizability; COI = conflict of interest. ✔ = low risk of bias; !!! = high risk of bias; ? = unclear risk of bias. NA = not applicable.

The risk of information bias in the ascertainment of the exposure was low in all but one study. Misclassification of the outcome was low in 10 studies, where PD was confirmed based on diagnostic codes in addition to the use of anti‐PD medication (e.g., levodopa), or was identified by neurologists or from the PD disease registry [32] (Figures 2 and 3). Most studies concerning β‐antagonists (*N* = 11/14) were at moderate or high risk of protopathic bias. Of the 22 studies, 9 did not control for confounding by socio‐demographic factors, and 15 did not consider the area of residence (urban/rural). Apart from 10 studies, all others were noted to have potential limitations due to the lack of control for confounding due to smoking. Eight studies did not investigate different time lags between exposure to the drugs and incidence of PD, or any dose–response. Most studies did not define a clear time window for patient exposure to study drugs, nor did they provide a clear rationale for selecting a specific time window, especially regarding the time considered at risk for developing PD or a putative protective effect of the β2‐agonists towards it. Generalisability was a concern for six studies, and all but two studies provided a clear declaration of conflict of interests.

### 3.3 Summary RR Estimates of the Association Between β‐Antagonists and Parkinson's Disease

The pooled effect estimates for the class of β‐antagonists was 1.41 (95% CI 1.18–1.68, I^2^ = 99.2%, *p* < 0.001, 12 studies) (Figure 2). The summary estimate for non‐selective β‐antagonists alone was 1.57 (95% CI: 1.14–2.15, I^2^ = 93.6%, *p* < 0.001, 3 studies). Summary estimates for individual non‐selective β‐antagonists drugs varied between 2.36 (95% CI: 1.66–3.36, I^2^ = 98.7%; 7 studies) for propranolol and 0.84 (95% CI 0.80–0.88, I^2^ = 0.0%, 3 studies) for carvedilol. Among selective β1‐antagonists, summary estimates found similar risks of PD among those exposed and unexposed to these drugs (1.00, 95% CI 0.95–1.04, I^2^ = 0.0%, 2 studies), with no meaningful differences for specific drugs.

### 3.4 Summary RR Estimates of the Association Between β2‐Agonists and Parkinson's Disease

Regarding β2‐agonists, the overall RR for the class was 0.93 (95% CI 0.84–1.03, I^2^ = 85.6%, *p* < 0.001, 11 studies) (Figure 3). The summary RR estimate for the two studies that looked at selective β2‐agonists was 0.90 (95% CI 0.79–1.02, I^2^ = 79.7%, *p* = 0.03). The studies that included only short‐acting β‐agonists showed a 9% lower risk of PD (0.91, 95% CI 0.88–0.94, I^2^ = 12.6%, *p* = 0.334), while the risk reduction was greater in studies of long‐acting β2‐agonists (0.85, 95% CI 0.76–0.96, I^2^ = 62.5%, *p* = 0.031) (Figure 3). Only two studies evaluated ultra‐long‐acting β2‐agonists, with summary RR estimates of 0.68 (95% CI: 0.49–0.94, I^2^ = 28.9%, *p* = 0.236) (Figure 3). Salbutamol was the most studied individual drug, with summary RR estimates of 0.88 (95% CI: 0.77–1.01, I^2^ = 91.9%, *p* < 0.001).

The studies that included only participants with COPD, asthma, and/or bronchiectasis showed similar patterns (Appendix S4).

For the class of β2‐agonists, in the study by de Germay et al. [27] those with diabetes had an increased risk of PD, while those without diabetes had a decreased risk of PD, which was the only interaction identified in this systematic review (Figure 3).

### 3.5 Dose and Duration Response Between Exposure and Outcome Considerations

In six studies [6, 23, 26, 27, 29, 36] there was an inverse association (decreasing in magnitude) between increased duration of use of the β‐antagonists and the risk of PD. Within studies undertaken by Mittal et al. [4] and Gronich et al. [5] propranolol continued to be significantly associated with increased risk of PD after 1–2 years and 2–8 years of use, with estimates decreasing in both studies for longer durations. Two studies reported no association with increasing duration of use of β2‐agonists [30, 36]. Giorgianni et al. [29] reported that the RR of 0.83 (95% CI: 0.75–0.91) with ever‐users of β2‐agonists was no longer observed after more than 2 years of cumulative duration of use (RR 0.97, 95% CI 0.80–1.17).

### 3.6 Sensitivity and Sub‐Group Analyses

For both β‐antagonists and β‐agonists, sensitivity analysis using the RR estimate for the longest period of exposure, when this was available, resulted in weaker associations but unchanged statistical significance. In studies of β‐antagonists, sub‐group analysis restricting on quality domains yielded similar associations to those in the main analysis, except when restricting to studies at low risk of protopathic bias where the results were no longer statistically significant. For β‐agonists, results were mostly unchanged (Table 2).

**TABLE 2:** Results from sensitivity and subgroup analyses. All estimates are from random effects meta‐analysis.

	No. of studies references	Summary effect size			Heterogeneity	
		RR	95% CI	*p*	I^2^ (%)	*p*
**β‐antagonists, all**						
Main analysis	12 [5, 22, 23, 24, 26, 27, 29, 33, 35, 36, 37, 38]	1.41	1.18–1.68	< 0.001	99.2	< 0.001
Low risk of bias in all domains	0	—	—	—	—	—
Only studies at low risk of outcome misclassification	4 [22, 27, 36, 37]	1.34	1.08–1.67	0.009	87.3	< 0.001
Only studies at low risk of protopathic bias	3 [27, 29, 33]	1.16	0.91–1.47	0.235	94.2	< 0.001
Only studies at low risk of confounding by SES	4 [5, 27, 33, 36]	1.20	0.98–1.48	0.08	91.9	< 0.001
Only studies at low risk of confounding by residence area	4 [5, 22, 27, 38]	1.21	1.07–1.37	0.002	574.0	0.073
Only studies at low risk of confounding by smoking	5 [22, 29, 33, 36, 38]	1.31	1.10–1.54	< 0.002	82.4	< 0.001
Only studies with time lag	8 [22, 23, 26, 27, 29, 33, 36, 37]	1.25	1.09–1.44	0.001	92.6	< 0.001
Considering the longest duration of exposure when provided	12 [5, 22, 23, 24, 26, 27, 29, 33, 35, 36, 37, 38]	1.34	1.10–1.64	0.003	99.3	< 0.001
**β‐antagonists, propranolol**						
Main analysis	7 [4, 5, 6, 24, 25, 27, 36]	2.36	1.66–3.36	< 0.001	98.7	< 0.001
Low risk of bias in all domains	0	—	—	—	—	—
Only studies at low risk of outcome misclassification	2 [27, 36]	3.05	1.57–5.95	0.009	88.1	0.004
Only studies at low risk of protopathic bias	2 [6, 27]	1.63	1.12–2.39	0.011	69.3	0.071
Only studies at low risk of confounding by SES	6 [4, 5, 6, 25, 27, 36]	2.13	1.50–3.03	< 0.001	96.9	< 0.001
Only studies at low risk of confounding by residence area	3 [5, 25, 27]	1.93	1.27–2.95	0.002	95.2	< 0.001
Only studies at low risk of confounding by smoking	1 [6]	1.41	1.27–1.56	< 0.001	—	—
Only studies with time lag	4 [6, 25, 27, 36]	2.01	1.18–3.42	0.010	97.3	< 0.001
Considering the longest duration of exposure when provided	7 [4, 5, 6, 24, 25, 27, 36]	1.74	1.17–2.58	0.006	98.4	< 0.001
**β2‐agonists, all**						
Main analysis	11 [5, 27, 28, 29, 31, 32, 34, 35, 36, 39, 40]	0.93	0.85–1.01	0.098	85.6	< 0.001
Low risk of bias in all domains	0	—	—	—	—	—
Only studies at low risk of outcome misclassification	7 [27, 28, 31, 32, 34, 36, 40]	0.97	0.82–1.15	0.765	85.5	< 0.001
Only studies at low risk of confounding by SES	8 [5, 28, 31, 32, 36, 39, 40]	0.92	0.81–1.03	0.159	86.7	< 0.001
Only studies at low risk of confounding by residence area	4 [5, 27, 28, 39]	0.96	0.79–1.18	0.721	74.3	0.004
Only studies at low risk of confounding by smoking	4 [28, 29, 39, 40]	0.95	0.83–1.07	0.383	69.3	0.021
Only studies with time lag	8 [5, 27, 28, 29, 31, 34, 36, 39]	0.92	0.80–1.06	0.272	73.8	< 0.001
Considering the longest duration of exposure when provided	11 [5, 27, 28, 29, 31, 32, 34, 35, 36, 39, 40]	0.95	0.87–1.02	0.130	74.1	0.001
**β2‐agonists, short‐acting**						
Main analysis	6 [5, 30, 31, 36, 39, 40]	0.91	0.88–0.94	< 0.001	12.6	0.334
Low risk of bias in all domains	0	—	—	—	—	
Only studies at low risk of outcome misclassification	3 [31, 36, 40]	0.93	0.84–1.03	0.175	55.6	0.105
Only studies at low risk of confounding by SES	6 [5, 30, 31, 36, 39, 40]	0.91	0.88–0.94	< 0.001	12.6	0.334
Only studies at low risk of confounding by residence area	2 [5, 39]	0.89	0.83–0.96	0.002	0.0	0.833
Only studies at low risk of confounding by smoking	3 [30, 39, 40]	0.94	0.86–1.02	0.121	46.2	0.156
Only studies with time lag	4 [5, 31, 36, 39]	0.90	0.85–0.94	< 0.001	0.0	0.709
**β2‐agonists, long‐acting**						
Main analysis	5 [5, 31, 36, 39, 40]	0.85	0.76–0.96	0.008	62.5	0.031
Low risk of bias in all domains	0	—	—	—	—	
Only studies at low risk of outcome misclassification	3 [31, 36, 40]	0.84	064–1.10	0.203	72.5	0.026
Only studies at low risk of confounding by SES	5 [5, 31, 36, 39, 40]	0.85	0.76–0.96	0.008	62.5	0.031
Only studies at low risk of confounding by residence area	2 [5, 39]	0.83	0.75–0.91	< 0.001	0.0	0.421
Only studies at low risk of confounding by smoking	2 [39, 40]	0.87	0.69–1.10	0.252	64.5	0.093
Only studies with time lag	4 [5, 31, 36, 39]	0.81	0.69–0.95	0.008	64.3	0.039
**β2‐agonists, salbutamol**						
Main analysis	7 [4, 5, 6, 24, 25, 27, 36]	0.88	0.77–1.01	0.066	91.9	< 0.001
Low risk of bias in all domains	0	—	—	—	—	
Only studies at low risk of outcome misclassification	3 [4, 27, 36]	0.77	0.61–0.98	0.037	59.2	0.061
Only studies at low risk of confounding by SES	6 [4, 5, 6, 25, 27, 36]	0.84	0.74–0.96	0.011	84.7	< 0.001
Only studies at low risk of confounding by residence area	3 [5, 25, 27]	0.86	0.81–0.92	< 0.001	47.2	0.128
Only studies at low risk of confounding by smoking	1 [6]	0.97	0.93–1.01	0.148	—	—
Only studies with time lag	4 [5, 6, 27, 36]	0.92	0.85–1.01	0.065	56.1	0.058

### 3.7 Publication Bias

**FIGURE 4:**
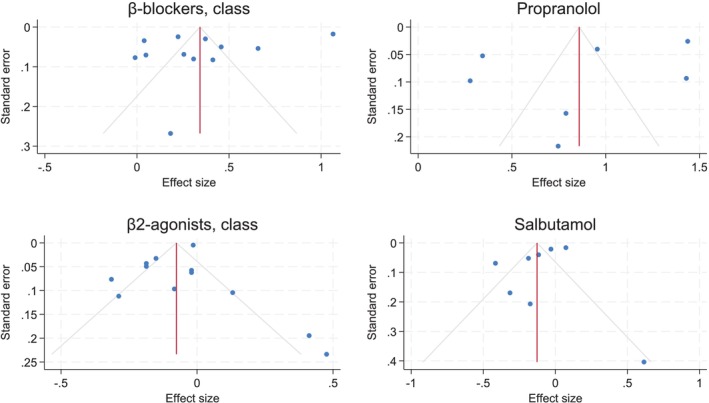
Funnel plots.

There was no evidence of publication bias in the studies of β‐antagonists, propranolol, and salbutamol (Figure 4). This was supported by the results of the Egger's test (*p* = 0.34 for the whole class; *p* = 0.60 for propranolol; *p* = 0.68 for salbutamol). The funnel plot for β2‐agonists appeared asymmetrical on visual inspection, and the Egger's test suggested possible publication bias (*p* = 0.03).

## 4 Discussion

### 4.1 Summary of Results

We identified 22 studies that provided data on the association between β‐AR drugs and risk of PD. Overall, ever users of β‐antagonists had a 41% increased risk of PD compared to never users of β‐antagonists. The summary effect estimates for different β‐antagonists provided divergent results. Propranolol was associated with a 2.36‐fold increased risk of PD, while carvedilol showed a 16% protective effect. The mechanism of this apparent protective effect of carvedilol could be hypothesised as due to its distinctive mechanism of action, namely the maintaining of cardiac output by decreasing afterload with a cardiac beta blockade. It has also additional antioxidant effects, reduced neutrophil infiltration, apoptosis inhibition, diminished vascular smooth muscle migration, among others [41]. For β2‐agonists, the meta‐analysis estimated a 7% non‐significant reduced risk of PD among ever users compared to never users. The same direction of effect was observed for individual β2‐agonist drugs, with significant protective effects for short, long‐ and ultra‐long acting β2‐agonists, as follows: 9% protective effect for short‐acting, 15% for long‐acting and 32% for ultra‐long acting. However, most studies were subject to important biases, in addition to publication bias for studies of β2‐agonists, which may entirely explain the associations. These biases are discussed in detail below.

### 4.2 Strengths and Limitations of the Included Studies, With Discussion of the Potential Sources of Bias and Their Impact

A strength of the studies included in this review is the low potential for misclassification of the exposure. Studies of electronic health records or prescription databases usually have high validity, particularly for prescriptions [42], though the validity of medication usage may be lower due to non‐adherence. This would tend to under ascertain any true protective or harmful association. Another strength is the longitudinal nature of the data used (recorded prospectively allowing incident disease recorded after exposure to be identified), which ensured temporality and allowed for the evaluation of different latency periods and/or time/dose of exposure.

Protopathic bias is the most important limitation of the studies of β‐antagonists included within the analysis. In analyses of propranolol (and consequently those for the whole class of β‐antagonists), reverse causality cannot be excluded in studies with a lack of, or suboptimal consideration of tremor, a bias likely to move results away from the null and overestimate the association. Propranolol is the main β‐antagonist indicated for the treatment of tremor, which may be an early sign of Parkinson's disease. Becker et al. [38] found an increased risk of Parkison's disease only in patients without recorded cardiovascular disease; a review of a random sample of records revealed a high proportion of patients with tremor symptoms. PD is also a complex diagnosis and may not always be coded correctly. Variations in the definitions of PD, for example in relation to inclusion of cases of parkinsonism, may limit comparability of studies. Some studies excluded patients with tremor recorded in electronic health records databases [4]; however, it is possible that tremor was underrecorded (seen as a sign, not a diagnosis), and the sensitivity of these exclusions suboptimal, particularly in studies of prescription databases that are not designed to capture morbidity. This was also supported by studies that tended to report an inverse association (decreasing in magnitude) between increased duration of use of the β‐blockers and the risk of Parkinson's disease. Mittal et al. [4] and Gronich et al. [5] judged their results as unlikely evidence of reverse causality since the associations remained significant several years after exposure, but these findings were not replicated in the study by Giorgianni et al. [29], that used UK primary care data. This study evaluated the rates of Parkison's disease within 1‐, 2‐, 3‐, and 5‐year latency windows of use of β‐antagonists, and showed that the rate was highest in year 1, decreased thereafter, and was no longer increased after 5 years, which supports that long‐term use is not associated with increased risk of PD. Finally, the protective results observed for carvedilol, a non‐selective brain–blood‐barrier crossing β‐blocker (like propranolol) without indication for tremor (contrary to propranolol), support the role of bias in the results due to lack of adjustment for smoking and other confounders.

Unmeasured and residual confounding by smoking was a significant limitation in several studies, as smoking is a protective factor in PD [43] and a major risk factor for cardiovascular disease (treated with β2‐antagonists), and asthma [44] and COPD [45] (treated with β2‐agonists). Adjustments for smoking generally attenuated the effect estimates, but residual confounding cannot be excluded, particularly in studies of prescription databases, where recording of smoking status is likely suboptimal, and in studies that used proxies of smoking such as comorbidities and socio‐economic status [5]. Studies that looked only at COPD/asthma patient populations [28, 30, 32, 39] were still likely affected by residual confounding by smoking, as not all COPD cases are from smoking, and even within an apparently homogeneous population, there would be a wide range of smoking intensity history [46]. Similarly, people with asthma are less likely to be smokers, and thus careful consideration is needed when interpreting results from studies that included people with asthma, COPD, and other respiratory diseases. One study [39] provided results stratified by COPD and asthma patients, and reported no dose–response association between increased use of β2‐agonists and PD. Recent studies have also suggested an association between migraine and PD [47]. β2‐antagonists are often first‐line preventive treatment in migraine. Migraine was seldom considered in the original studies, except in the studies by Gronich et al. [5] and De Germay et al. [27].

The generalisability of the studies that included patients with asthma and COPD is also limited, as these are unlikely to represent the general population in terms of life‐long risk factors as well as concomitant medications (e.g., use of β‐agonists and anticholinergics).

### 4.3 Comparison With Other Studies

Two previous systematic reviews/meta‐analysis [48, 49] investigated the same associations and concluded that β‐antagonists were associated with increased risk of PD and β‐agonists with protective effect. These reviews identified fewer studies (10 and 15, respectively) and did not provide a risk of bias assessment. Hopfner et al. [50] conducted a ‘quick review’ of six studies on this topic and found similar results for β‐antagonists and β‐agonists, again without bias assessment. To our knowledge, the current study is the most comprehensive and up to date systematic review of published studies, including 22 studies published between 2007 and 2024, including a rigorous bias assessment and discussion of the implications. A review of prospective cohort studies on the possible risk/protective factors underlying the development, progression and clinical subtypes of PD, found that only a few of them are based on epidemiological evidence and some biological plausibility [51]. We found similar summary RR estimates to previous reviews but, in a conservative approach, we suggest that the available evidence is not unequivocal because it is possible that the reported associations are due to bias and confounding, rather than a causal association.

### 4.4 Strengths and Limitations of the Current Review

This systematic review was based on the extensive search of two major established databases. Although unlikely, it is possible that some papers may not have appeared in our database searches, for example, if the keywords did not appear in the title and abstract, or due to inaccurate indexing in the publications database. We additionally reviewed the references of other studies in the topic to improve sensitivity and included conference abstracts to minimise the risk of important results being missed. Other strengths of this review included the a priori definition of the methods (see PROSPERO protocol) and systematic approach to study selection and data extraction, with duplicate screening of studies using pre‐defined inclusion/exclusion criteria. Efforts were also made to group drugs belonging to each class accurately; however, considering the non‐consistent nomenclature used across the reviewed studies and lack of access to the primary data, it cannot be excluded that some results have been misclassified. Study authors were contacted for additional information to clarify relevant aspects.

### 4.5 Implications for Clinical Practice

Drugs in the β‐antagonist class are valid treatment options in a range of cardiovascular indications [7]. Even if the studies were unbiased, the strength of the associations reported would not support any implications for clinical practice. Hopfner et al. [50] estimated that, if the association was causal, the absolute risk of PD associated with 5 years of propranolol use was one case in 10 000 individuals exposed, which would be considered a very rare adverse effect and would not outweigh the known benefits of treatment. Using salbutamol for PD prevention would require treating 50 000 people for 5 years to prevent one case of PD [50].

### 4.6 Implications for Future Research

Our review shows a need for carefully designed population‐based studies, with low potential for protopathic bias (e.g., excluding symptoms of tremor in primary care databases), carefully chosen lag times, adjustment for important confounders such as socio‐economic status, area of residence, smoking, and consideration of exposure drugs indication (e.g., migraine). The risk of PD associated with β2‐agonists with different durations of action (SABA, LABA and ultra‐LABA) has been seldom studied due to the limited number of research studies to date and could be the focus of further studies to better understand the role of publication bias in the results of this review.

## 5 Conclusion

The available epidemiologic data are not sufficiently consistent to infer a causal relationship between the use of β‐antagonists and an increased risk of PD. As regards to the β2‐agonists class, there was a reduction in the risk of PD, consistent in direction and showing stronger effects for β2 agonists with longer duration of action. Currently it is unknown how much of the small protective effect observed could be due to unmeasured confounding by smoking or publication bias.

### 5.1 Plain Language Summary

β‐agonists and β‐antagonists are widely prescribed medications for conditions like asthma and heart disease. In 2018, concerns were raised about a potential link between propranolol, a β‐antagonist, and Parkinson's disease (PD). This prompted an investigation by European regulators, which found no need for changes to product safety information. Since then, more studies have explored this potential connection. We systematically reviewed and analysed 22 studies examining whether β‐antagonists (like propranolol) or β‐agonists affect the risk of developing PD. Most studies had significant limitations, including biases related to how outcomes were classified, how patients were selected, and how factors like smoking or socio‐economic status were accounted for. For propranolol, a key issue is “protopathic bias,” as it is often used to treat essential tremor, a condition that may be mistaken for early PD. Our analysis found that β‐antagonists, particularly propranolol, were associated with a higher risk of PD, while β2‐agonists showed no strong link or even a potential protective effect. However, when we focused on higher‐quality studies, these associations became weaker or disappeared. Overall, the evidence does not support a causal link between these medications and PD. The observed associations are likely due to biases and confounding factors rather than a real effect.

## Conflicts of Interest

The authors declare no conflicts of interest.

## Supporting information


Data S1.
