# Supplementary material for: Association Between β‐Adrenoreceptor Agonists and Antagonists and Parkinson's Disease: Systematic Review and Meta‐Analysis
Source: Pharmacoepidemiol Drug Saf. 2025 Apr 9;34(4):e70140. doi: 10.1002/pds.70140 (PMC11979683; doi:10.1002/pds.70140)
Supplement: Supplementary file 1 — Data S1. [file PDS-34-e70140-s001.docx]

**Supplementary file**

**Paper:** Association between β-adrenoreceptor agonists and antagonists and Parkinson’s disease: systematic review and meta-analysis

**Authors:** Agnieszka Szmigiel, Miguel Monteiro da Rocha, Kate Browne, Daniel Morales, David Benee Olsen, Charlotte Warren-Gash, Ian Douglas, Krishnan Bhaskaran, Helena Carreira

## Appendix 1. Medline search expression.

| 1 | exp Parkinsonian Disorders |
| --- | --- |
| 2 | Parkinson*.mp. |
| 3 | 1 or 2 |
| 4 | exp Adrenergic beta-Antagonists/ |
| 5 | (beta* adj3 (adrenergic* or adrenoreceptor* or agonist* or antagonist* or block* or receptor*)).mp. [mp=title, abstract, original title, name of substance word, subject heading word, floating sub-heading word, keyword heading word, organism supplementary concept word, protocol supplementary concept word, rare disease supplementary concept word, unique identifier, synonyms] |
| 6 | ((beta* adj6 (long?acting or LABA or SABA or ultra?LABA or short?acting)) and agonist*).mp. [mp=title, abstract, original title, name of substance word, subject heading word, floating sub-heading word, keyword heading word, organism supplementary concept word, protocol supplementary concept word, rare disease supplementary concept word, unique identifier, synonyms] |
| 7 | acebutolol/ or alprenolol/ or atenolol/ or betaxolol/ or bisoprolol/ or bupranolol/ or carvedilol/ or carteolol/ or celiprolol/ or labetalol/ or metipranolol/ or metoprolol/ or nadolol/ or oxprenolol/ or penbutolol/ or pindolol/ or practolol/ or prenalterol/ or propranolol/ or sotalol/ or timolol/ or xamoterol/ |
| 8 | salbutamol/ or terbutaline/ or fenoterol/ or hexoprenaline/ or isoetarine/ or pirbuterol/ or procaterol/ or tretoquinol/ or carbuterol/ or tulobuterol/ or bambuterol/ or clenbuterol/ or reproterol/ or terbutaline/ or clenbuterol/ or albuterol/ |
| 9 | bitolterol/ or fenoterol/ or hexoprenaline/ or isoprenaline/ or isoproterenol/ or levosalbutamol/ or levalbuterol/ or orciprenaline/ or metaproterenol/ or pirbuterol/ or procaterol/ or terbutaline/ or isoxsuprine/ or ritodrine/ or carbuterol/ or isoetarine/ or reproterol/ or rimiterol/ or tulobuterol/ |
| 10 | arformoterol/ or bambuterol/ or clenbuterol/ or formoterol/ or salmeterol/ or abedirol/ or carmoterol/ or indacaterol/ or milveterol/ or olodaterol/ or vilanterol/ |
| 11 | exp Adrenergic beta-Agonists/ |
| 12 | 4 or 5 or 6 or 7 or 8 or 9 or 10 or 11 |
| 13 | 3 and 12 |

## Appendix 2. Criteria used to judge the risk of bias of the studies included in the systematic review.

| **Judgment of risk** | **Ascertainment of exposure (βAR drugs)** | **Ascertainment of outcome**  **(Parkinson’s disease)** | **Protopathic  bias^1^** | **Control for confounding by socio-demographic factors^2^** | **Control for**  **confounding by urban/rural residence^2^** | **Control for confounding by smoking^3^** | **Evaluation of dose or duration of exposure and response** | **Time lag consideration** | **Generalisability** | **Conflict of interest** |
| --- | --- | --- | --- | --- | --- | --- | --- | --- | --- | --- |
| **Low risk of bias** | From pharmacy records  OR  prescription database | Clinical diagnoses, ICD or other codes for PD specified  AND  Review of cases or other confirmatory method (e.g., prescription of levodopa)  OR  Verified by neurologists  OR  Sourced from PD registry | Tremor adjusted for in analysis  OR  Comparison of models adjusted and unadjusted for tremor  OR  Sub-analysis restricting to β-antagonists not indicated for tremor | Adjusted for sex, age, socio-economic status (e.g. level of education) | Adjusted for in analysis | Adjusted for a measure of smoking in analysis | Lag provided by different time lags and/or cumulative doses | Lag provided by different time lags | General population database | The study authors explicitly report the existence, or not, of conflicts of interests  AND  the study funders are acknowledged |
| **Moderate / unclear risk of bias** | Self-reported intake  OR  Not reported | ICD or other codes for PD without further checks | Attempted to exclude patients with history of tremor | Not mentioned | Adjusted for a proxy (e.g., GP practice) | Adjusted for a proxy of smoking (e.g., COPD) | Single time lag/ dosage | Single time lag | Selected groups of the population (e.g., certain ethnicities excluded) | Not applicable |
| **High risk of bias** | Not mentioned | Definition or codes not mentioned | No accounting for tremor in patient selection and/or analysis  AND  No sub analysis for β-antagonists not indicated to treat tremor | Adjusted for one or more, but not all, of the following: sex, age, socioeconomic status | Lack of consideration | Lack of consideration | Lack of consideration for duration or dose of exposure | Lack of consideration | Selection criteria not provided | The presence or absence of conflicts of interest is not reported  AND  No study funding is acknowledged |

^1^ Some β-antagonists (e.g. propranolol) have been used for decades to treat tremor, which is an early sign of Parkinson’s disease.

^2^ The literature indicates that (older) age, (male) sex, (lower) socio-economic status, and area of residence (rural) area associated with increased risk of Parkinson’s disease.

^3^ Tobacco smoking is causally associated with a decreased risk of Parkinson’s disease (Mappin-Kasirer B, Neurology 2020;94:e2132-e2138).

## Appendix 3. Characteristics of the individual studies.

|  | **First author,**  **publication year,**  **country of data** | **Study design,**  **Database,**  **Years of study data** | **Number of participants, mean/median age (SD), % of men** | **Definition of exposure (drug / drug class)** | **Study comparisons** | **Control for confounding** | **Study results** |
| --- | --- | --- | --- | --- | --- | --- | --- |
| 1. | Ton et al., 2007  United States | Population based nested case-control study  GHC database  1992 - 2002 | Cases: 206 incident cases of PD, 69.2 (9.0), 58.7%  Controls: 383 individuals without PD, 69.4 (8.6), 62.4% | At least two prescriptions of β-antagonists filled up to 5 years prior to the study | Ever vs. never use  Cumulative duration of use  Cumulative standard doses  Average daily standard doses  Total number of prescriptions  Pattern of use | Matched on age, sex, duration of enrolment in database, and clinic. | **β-antagonists, whole class**  **(5-year time lag)**  Ever use vs. never use:  OR=1.20 (95% CI: 0.71-2.03)  Cumulative duration of use:  <3 years vs. no use:  OR=1.23 (0.65-2.30)  ≥3 years vs. no use:  OR=1.16 (0.54-2.52)  Cumulative standard doses:  <4300 vs. no use:  OR=1.38 (0.65–2.84)  ≥4300 vs. no use:  OR=1.07 (0.55–2.11)  Average daily standard doses:  <4 vs. no use:  OR= 1.39 (0.72–2.66)  >=4 vs. no use:  OR=0.99 (0.47–2.09)  Total number of prescriptions:  <20 vs. no use:  OR=1.37 (0.72–2.62)  ≥20 vs. no use:  OR=1.00 (0.48–2.11)  Pattern of use:  Intermittent use vs. no use:  OR=0.92 (0.35–2.41)  Continuous for at  least 6 months vs. no use:  OR=1.17 (0.58–2.33)  *(continues)* |
| 1. | Ton et al., 2007  United States | Population based nested case-control study  GHC database  1992 - 2002 | Cases: 206 incident cases of PD, 69.2 (9.0), 58.7%  Controls: 383 individuals without PD, 69.4 (8.6), 62.4% | At least two prescriptions of **propranolol or metoprolol** up to 5 years before the date of the study | Ever vs. never use  Cumulative duration of use  Cumulative standard doses  Average daily standard doses  Total number of prescriptions | Age, sex, smoking, clinic, duration of clinic enrolment, heart disease. | *(continued)*  **Propranolol or metoprolol**  **(5-year time lag)**  Ever use vs. never use:  OR=1.47 (95% CI: 0.80-2.69)  Cumulative duration of use:  <9 months vs. no use:  OR=2.15 (0.94–4.94)  ≥9 months vs. no use:  OR=1.05 (0.47–2.35)  Cumulative standard doses:  <885 vs. no use:  OR=1.77 (0.77–4.07)  ≥885 vs. no use:  OR=1.25 (0.56–2.78)  Average daily standard doses:  <4 vs. no use:  OR= 1.72 (0.76–3.90)  >=4 vs. no use:  OR= 1.26 (0.55–2.86)  Total number of prescriptions:  <10 vs. no use:  OR= 2.16 (0.94–4.94)  ≥10 vs. no use:  OR= 1.04 (0.46–2.34) |
| 2. | Becker et al., 2008  United Kingdom | Population-based nested case-control study (≥40 years old)  GPRD  1994 - 2005 | Cases: 3,637 incident PD cases, NP, 59.6%  Controls: 3,637 individuals without PD, NP, 59.6% | β-antagonists | Past use vs. non-use  Current use vs. non-use  Number of prescriptions | Matched on age, sex, general practice, index date, and database duration of enrolment.  Further adjusted for BMI, smoking, comorbidities, and use of diuretics and statins. | **Main analysis:**  Past use vs. non-use:  OR= 1.36 (95% CI: 1.16–1.59)  Current use vs. non-use:  OR= 1.19 (95% CI: 1.02–1.39)  Number of prescriptions:  1-9 vs. non-use:  OR= 1.79 (1.29–2.48)  10-29 vs. non-use:  OR= 0.91 (0.70–1.19)  ≥30 vs. non-use:  OR= 1.16 (0.95–1.41)  **Restricting to patients with history of CVD:**  Current use vs. non-use:  OR= 0.99 (95% CI: 0.55-1.76)  **Restricting to patients with no history of CVD:**  Current use vs. non-use:  OR= 8.86 (95% CI: 2.97-26.46) |
| 3 | Ritz et al., 2010  Denmark | Population-based matched case-control  Danish Hospital Register linked to the Registry of Medical Products Statistic and Central Population Registry  2001-2006 | Cases: 1,931 incident PD cases, 72.2 (10.5), 58.1%  Controls: 9,651 individuals without PD, 72.2 (10.5), 58.1% | ≥2 prescriptions of β-antagonists (ATC group C07A), filled at least ≥2 years prior to index date | ≥2 prescriptions vs. never use | Matched on age and sex.  Further adjusted for age, sex, chronic obstructive pulmonary disease (5-year lag), Charlson index (5-year lag). | **2-year time lag**  ≥2 prescriptions vs. never use:  OR= 1.29 (95% CI: 1.13-1.48)  **5-year time lag**  ≥2 prescriptions vs. never use:  OR= 1.16 (0.99-1.37) |
| 4. | Mittal et al., 2017  Norway | Population-based cohort study  NorPD  2004-2007 | Cohort of 4,487,059  Follow up 11 years  PD incident cases in never/ever users of propranolol:  4593/41  PD incident cases in never/ever users of salbutamol:  4398/236  58.3% | Propranolol  Salbutamol | Ever vs. never use | Adjusted for age, sex, education level | **Propranolol**  Ever use vs. never use:  1-year lag  RR= 2.20 (95% CI: 1.62-3.00)  2-year lag  RR = 1.82 (95% CI: missing)  **Salbutamol**  Ever use vs. never use:  RR=0.66 (0.58-0.76)  Cumulative standard doses:  <60 DDD vs. never use:  RR=0.96 (0.76-1.23)  60-180 DDD vs. never use:  RR=0.60 (0.40-0.91)  >180 DDD vs. never use:  RR=0.45 (0.31-0.67) |
| 5. | Gronich et al., 2018  Israel | Nested case-control study within a cohort of 1,762,164 individuals  CHS  2004 - 2017 | Cases: 11,314 incident PD cases, 74.7 (11.0), 57.6%  Controls: 113,140 individuals without PD, 74.6 (11.0), 57.6% | β-antagonists;  Non-selective β-antagonists (propranolol, carvedilol, labeterol, pindolol, sotalol);  β1-selective (atenolol, bisoprolol, metoprolol);  atenolol; bisoprolol; carvedilol; formoterol; metoprolol; propranolol; satolol.  β2-agonists; SABA (salbutamol, terbutaline);  LABA (salmeterol, salbutamol);  ultra-LABA (vilanterol, olodaterol and indacaterol);  salbutamol; salmeterol;  terbutaline; vilanterol. | Ever vs. never use  Number of prescriptions  Different time lag | Matched on age, sex, ethnic group and duration of follow up.  Adjusted for asthma, COPD, ischaemic heart disease, congestive heart failure, diabetes mellitus, hypertension, migraine, smoking, alcohol,  aspirin, ibuprofen, statins, valproate, calcium channel antagonists,  use of β2-agonists and β- antagonists | **β-antagonists, whole class**  Ever use vs. never use:  RR=1.25 (1.19-1.31)  **Non-selective β-antagonists**  Ever use vs. never use:  RR=2.04 (1.90-2.20)  **Selective β1-antagonists**  Ever use vs. never use:  RR=1.00 (0.95-1.05)  **Propranolol**  Ever use vs. never use:  RR=2.60 (2.40-2.81)  Number of prescriptions:  2-year lag  <6 vs. never use:  RR=1.75 (1.54-2.00)  ≥6 vs. never use:  RR=2.50 (2.15-2.90)  5-year lag  <6 vs. never use:  RR=1.31 (1.08-1.58)  ≥6 vs. never use:  RR=1.89 (1.53-2.33)  8-year lag  <6 vs. never use:  RR=1.35 (1.03-1.78)  ≥6 vs. never use:  RR=1.42 (1.02-1.98)  **Atenolol**  Ever use vs. never use:  RR=0.97 (95% CI: 0.92-1.02)  **Bisoprolol**  Ever use vs. never use:  RR=1.08 (0.99-1.17)  *(continues)* |
| 5. | Gronich et al., 2018  Israel | Nested case-control study within a cohort of 1,762,164 individuals  CHS  2004 to 30 June 2017 | Cases: 11,314 incident PD cases, 74.7 (11.0), 57.6%  Controls: 113,140 individuals without PD, 74.6 (11.0), 57.6% | β-antagonists;  non-selective β- antagonists;  β1-selective;  atenolol; bisoprolol; carvedilol; formoterol; metoprolol; propranolol; satolol.  β2-agonists; SABA;  LABA; salbutamol; salmeterol;  terbutaline; vilanterol. | Not reported | Matched on age, sex, ethnic group and duration of follow up.  Adjusted for asthma, COPD, ischaemic heart disease, congestive heart failure, diabetes mellitus, hypertension, migraine, smoking, alcohol,  aspirin, ibuprofen, statins, valproate, calcium channel antagonists,  use of β2-agonists and β-antagonists | *(continued)*  **Carvedilol**  Ever use vs. never use:  RR=0.86 (0.55-1.34)  **Metoprolol**  Ever use vs. never use:  RR=0.96 (0.85-1.07)  **Sotalol**  Ever use vs. never use:  RR=0.86 (0.55-1.34)  **Formoterol**  Ever use vs. never use:  RR=0.84 (0.74-0.95)  **Salbutamol**  Ever use vs. never use:  RR=0.89 (0.82-0.96)  Number of prescriptions:  <6 vs. never use:  RR=0.95 (0.83-1.08)  ≥6 vs. never use:  RR=0.65 (0.45-0.94)  **Salmeterol**  Ever use vs. never use:  RR=0.89 (0.79-1.00)  **Terbutaline**  Ever use vs. never use:  RR=0.87 (0.73-1.04)  **Vilanterol**  Ever use vs. never use:  RR=0.40 (0.17-0.91)  *(continues)* |
| 5 | Gronich et al., 2018  Israel | Nested case-control study within a cohort of 1,762,164 individuals  CHS  2004 to 30 June 2017 | Cases: 11,314 incident PD cases, 74.7 (11.0), 57.6%  Controls: 113,140 individuals without PD, 74.6 (11.0), 57.6% | β- antagonists;  non-selective β-antagonists;  β1-selective;  atenolol; bisoprolol; carvedilol; formoterol; metoprolol; propranolol; sotalol.  β2-agonists; SABA;  LABA; salbutamol; salmeterol;  terbutaline; vilanterol. | Not reported | Matched on age, sex, ethnic group and duration of follow up.  Adjusted for asthma, COPD, ischaemic heart disease, congestive heart failure, diabetes mellitus, hypertension, migraine, smoking, alcohol,  aspirin, ibuprofen, statins, valproate, calcium channel antagonists,  use of β2-agonists and β-antagonists | *(continued)*  **SABA**  Ever use vs. never use:  RR=0.89 (0.82-0.96)  **LABA**  Ever use vs. never use:  RR=0.84 (0.76-0.93)  **Ultra LABA**  Ever use vs. never use:  RR=0.49 (0.25-0.92)  **Excluding essential tremor**  ***β-antagonists***  **Non-selective**  Ever use vs. never use:  RR=1.53 (1.40-1.67)  **β1-selective**  Ever use vs. never use:  RR=1.02 (0.97-1.08)  **Propranolol**  Ever use vs. never use:  RR=1.90 (1.72-2.09)  ***Β2-agonists***  **Salbutamol**  Ever use vs. never use:  RR=0.87 (0.80-0.95)  **Formoterol**  Ever use vs. never use:  RR=0.79 (0.69-0.90)  **Vilanterol**  Ever use vs. never use:  RR=0.39 (0.16-0.96)  *(continues)* |
| 5 | Gronich et al., 2018  Israel | Nested case-control study within a cohort of 1,762,164 individuals  CHS  2004 to 30 June 2017 | Cases: 11,314 incident PD cases, 74.7 (11.0), 57.6%  Controls: 113,140 individuals without PD, 74.6 (11.0), 57.6% | β-antagonists;  non-selective β- antagonists;  β1-selective;  atenolol; bisoprolol; carvedilol; formoterol; metoprolol; propranolol; satolol.  β2-agonists; SABA;  LABA; salbutamol; salmeterol;  terbutaline; vilanterol. | Not reported | Matched on age, sex, ethnic group and duration of follow up.  Adjusted for asthma, COPD, ischaemic heart disease, congestive heart failure, diabetes mellitus, hypertension, migraine, smoking, alcohol,  aspirin, ibuprofen, statins, valproate, calcium channel antagonists,  use of β2-agonists and β-antagonists | *(continued)*  **SABA**  Ever use vs. never use:  RR=0.87 (0.80-0.95)  **LABA**  Ever use vs. never use:  RR=0.83 (0.74-0.92)  **Ultra-LABA**  Ever use vs. never use:  RR=0.46 (0.22-0.93) |
| 6. | Nielsen et al., 2019  United States | Population-based case-control study  Medicare  patients aged 66-90  2004–2009 | Cases: 48,295 incident PD cases, 78.6 (NP), NP  Controls: 52,324 individuals without PD, 76.4 (NP), NP | Carvedilol, metoprolol, propranolol, salbutamol. | Ever vs. never use in 2008-2009 | Adjusted for age, sex, race, smoking, oxygen therapy, nicotine and/or varenicline, overall use of medical in 2004-2009 (prior to diagnosis). | **Propranolol**  **Defined daily dose**  40-79 vs <40:  OR=0.94 (0.74-1.20  >79 vs <40:  OR=0.79 (0.63-0.99)  **Time lag**  No lag  Ever vs. never:  OR=1.41 (1.27-1.56)  6-month lag  Ever vs. never:  OR=1.20 (1.07-1.34)  1-year lag  Ever vs. never:  OR=1.11 (0.98-1.25)  1.5-year lag  Ever vs. never:  OR=0.97 (0.80-1.18)  **Excluding essential tremor from model**  No lag  Ever vs. never:  OR=3.62 (3.31-3.96)  6-month lag  Ever vs. never:  OR=3.04 (2.76-3.34)  1-year lag  Ever vs. never:  OR=2.70 (2.44-3.00)  1.5-year lag  Ever vs. never:  OR=2.24 (1.90-2.64)  *(continues)* |
| 6. | Nielsen et al., 2019  United States | Case-control study  Medicare  patients aged 66-90  2004–2009 | Cases: 48,295 incident PD cases, 78.6 (NP), NP  Controls: 52,324 individuals without PD, 76.4 (NP), NP | Carvedilol, metoprolol, propranolol, salbutamol. | Ever vs. never use in 2008-2009 | Adjusted for age, sex, race, smoking, oxygen therapy, nicotine and/or varenicline, overall use of medical in 2004-2009 (prior to diagnosis) | *(continued)*  **Carvedilol**  Defined daily dose  12.5-24 vs <12.5:  OR=0.94 (0.82-1.08)  >24 vs <12.5:  OR=0.76 (0.67-0.86)  Time lag  No lag  Ever vs. never:  OR=0.83 (0.78-0.87)  6-month lag  Ever vs. never:  OR=0.84 (0.79-0.90)  1-year lag  Ever vs. never:  OR=0.85 (0.79-0.91)  1.5-year lag  Ever vs. never:  OR=0.88 (0.78-0.98)  **Excluding essential tremor from model**  No lag  Ever vs. never:  OR=0.77 (0.73-0.81)  6-month lag  Ever vs. never:  OR=0.79 (0.74-0.83)  1-year lag  Ever vs. never:  OR=0.79 (0.74-0.84)  1.5-year lag  Ever vs. never:  OR=0.82 (0.74-0.92)  *(continues)* |
| 6. | Nielsen et al., 2019  United States | Case-control study  Medicare  patients aged 66-90  2004–2009 | Cases: 48,295 incident PD cases, 78.6 (NP), NP  Controls: 52,324 individuals without PD, 76.4 (NP), NP | Carvedilol, metoprolol, propranolol, salbutamol. | Ever vs. never use in 2008-2009 | Adjusted for age, sex, race, smoking, oxygen therapy, nicotine and/or varenicline, overall use of medical care in 2004-2009 (prior to diagnosis), and tremor. | *(continued)*  **Metoprolol**  Defined daily dose  50 vs <50:  OR=0.89 (0.83-0.96)  >50 vs <50:  OR=0.84 (0.79-0.90)  Time lag  No lag  Ever vs. never:  OR=0.94 (0.91-0.97)  6-month lag  Ever vs. never:  OR=0.95 (0.92-0.99)  1-year lag  Ever vs. never:  OR=0.94 (0.91-0.98)  1.5-year lag  Ever vs. never:  OR=0.94 (0.89-0.99)  **Excluding essential tremor from model**  No lag  Ever vs. never:  OR=0.94 (0.91-0.97)  6-month lag  Ever vs. never:  OR=0.95 (0.92-0.99)  1-year lag  Ever vs. never:  OR=0.94 (0.91-0.98)  1.5-year lag  Ever vs. never:  OR=0.94 (0.89-0.99)  *(continues)* |
| 6. | Nielsen et al., 2019  United States | Case-control study  Medicare  patients aged 66-90  2004–2009 | Cases: 48,295 incident PD cases, 78.6 (NP), NP  Controls: 52,324 individuals without PD, 76.4 (NP), NP | Carvedilol, metoprolol, propranolol, salbutamol. | Ever vs. never use in 2008-2009 | Adjusted for age, sex, race, smoking, oxygen therapy, nicotine and/or varenicline, overall use of medical care in 2004-2009 (prior to diagnosis), and tremor. | *(continued)*  **Salbutamol**  Ever vs. never:  OR=0.97 (0.93-1.01)  Defined daily dose  60-180 vs <60:  OR=1.09 (0.99-1.21)  >180 vs <50:  OR=0.98 (0.89-1.08)  **Salbutamol via a metered dose inhaler**  Ever vs. never:  OR=0.81 (0.77-0.84) |
| 7. | Stacey et al., 2018  United States | Cohort study    TriNetX Analytics  NR | 1,063,038 people exposed to salbutamol, 54 (NR), 39%  76,142 people exposed to propranolol, 52 (NR), 37%  Comparison group: people exposed to paracetamol  Follow up 20 years | Salbutamol, Propranolol | Salbutamol vs. paracetamol  Propranolol vs. paracetamol | Not reported | **Salbutamol**  Ever use vs. never use:  RR=1.08 (1.04-1.11)  **Propranolol**  Ever use vs. never use:  RR=4.22 (4.00-4.44)  **β-antagonists,**  Ever use vs. never use:  **RR 2.9 (2.8-3.00)**  **β-agonists,**  Ever use vs. never use:  **RR 0.95 (0.95-0.99)** |
| 8. | Cepeda et al., 2019  United States | Self-controlled cohort  Claims databases:  Optum, CCAE, MDCR, MDCD  June 2000 to April 2018 | Cohort of 117,015,066 people across 4 databases | β-antagonists (incl. propranolol carvedilol, and metoprolol);  β-antagonists, non-selective;  β-antagonists, selective;  Carvedilol;  Metoprolol; Propranolol;  Selective β2-adrenoreceptor  agonists;  Albuterol (Salbutamol) | Periods of exposure to the drugs vs. non-exposure | Yes, for time invariant confounding, by design (self-controlled) | **β-antagonists, non-selective**  Period exposed vs. period unexposed:  RR=1.24 (1.05-1.46)  **β-antagonists, selective**  Period exposed vs. period unexposed:  RR=0.99 (0.90-1.10)  **Selective β2-adrenoreceptor**  **agonists**  Period exposed vs. period unexposed:  RR=0.83 (0.75-0.93)  **Albuterol (Salbutamol)**  Period exposed vs. period unexposed:  RR=0.83 (0.75-0.92)  **Carvedilol**  Period exposed vs. period unexposed:  RR=0.86 (0.74-1.0)  **Metoprolol**  Period exposed vs. period unexposed:  RR=0.94 (0.89-0.99)  **Propranolol**  Period exposed vs. period unexposed:  RR=1.32 (1.09-1.60) |
| 9. | Hopfner et al., 2019  Denmark | Matched case-control  study  Linked data from Danish National Registries  1 Jan 2000 to 31 Dec 2012 | Cases: 2,790 incident PD cases, median 73 years (IQR 66-79), 58.7%  Controls: 11,160 individuals without PD, median 73 year  (IQR 67-79), 58.7% | β-antagonists;  β2-agonists;  SABA;  LABA;  atenolol, bisoprolol, formoterol, metoprolol, propranolol,  salbutamol, salmeterol, sotalol,  terbutaline. | Ever vs. never use  Long-term use (>3 years) vs. never use  Cumulative use in years | Matched on age and sex.  Adjusted for antipsychotics, statins, myocardial infarction, non-steroid anti-inflammatory drugs, asthma, stroke, depression, alcohol-related diseases, anxiolytics, hypnotics, sedatives, selective serotonin reuptake inhibitors, Charlson comorbidity index, education. | **β-antagonists**  Ever vs. never use:  OR=1.58 (1.43-1.74)  >3 years use vs. never use:  OR=1.28 (1.10-1.47)  0-1 year use vs. never use:  OR=1.97 (1.70-2.28)  1-3 years use vs. never use:  OR=1.53 (1.27-1.85)  3-5 years use vs. never use:  OR=1.33 (1.06-1.67)  5-8 years use vs. never use:  OR=1.41 (1.12-1.76)  8+ years use vs. never use:  OR=1.17 (0.91-1.50)  **Propranolol**  Ever vs. never use:  OR=4.18 (3.48-5.02)  >3 years vs. never use:  OR=2.26 (1.48-3.46)  0-1 year vs. never use:  OR=5.14 (4.06-6.50)  1-3 years vs. never use:  OR=4.21 (2.85-6.22)  3-5 years vs. never use:  OR=2.95 (1.64-5.30)  5-8 years vs. never use:  OR=2.10 (0.91-4.85)  8+ years vs. never use:  OR=1.48 (0.58-3.82)  *(continues)* |
| 9. | Hopfner et al., 2019  Denmark | Matched case-control  study  Linked data from Danish National Registries  2000- 2012 | Cases: 2,790 PD cases, median 73 years (NP), 58.7%  Controls: 11,160 individuals without PD, NP (NP), 58.7% | β- antagonists;  β2-agonists;  SABA;  LABA;  atenolol, bisoprolol, formoterol, metoprolol, propranolol,  salbutamol, salmeterol, sotalol,  terbutaline. | Ever vs. never use  Long-term use (>3 years) vs. never use  Cumulative use in years | Adjusted for antipsychotics, statins, myocardial infarction, non-steroid anti-inflammatory drugs, asthma, stroke, depression, alcohol-related diseases, anxiolytics, hypnotics, sedatives, SSRIs, Charlson comorbidity index, education. | *(continued)*  **Atenolol**  >3 years vs. never use  OR=1.04 (0.73-1.49)  **Bisoprolol**  >3 years vs. never use  OR=0.67 (0.38-1.19)  **Metoprolol**  >3 years vs. never use  OR=1.35 (1.12-1.62)  **Sotalol**  >3 years vs. never use  OR=1.29 (0.76-2.19)  **β2-agonists**  Ever vs. never use:  OR=0.73 (0.63-0.85)  >3 years vs. never use:  OR=0.64 (0.42-0.98)  0-1 year vs. never use:  OR=0.69 (0.57-0.82)  1-3 years vs. never use:  OR=0.96 (0.70-1.31)  3-5 years vs. never use:  OR=0.70 (0.40-1.22)  5-8 years vs. never use:  OR=0.59 (0.30-1.18)  8+ years vs. never use:  OR=0.64 (0.28-1.42)  *(continues)* |
| 9. | Hopfner et al., 2019  Denmark | Matched case-control  study  Linked data from Danish National Registries  2000- 2012 | Cases: 2,790 PD cases, median 73 years (NP), 58.7%  Controls: 11,160 individuals without PD, NP (NP), 58.7% | β-antagonists;  β2-agonists;  SABA;  LABA;  atenolol, bisoprolol, formoterol, metoprolol, propranolol,  salbutamol, salmeterol, sotalol,  terbutaline. | Ever vs. never use  Long-term use (>3 years) vs. never use  Cumulative use in years | Adjusted for antipsychotics, statins, myocardial infarction, non-steroid anti-inflammatory drugs, asthma, stroke, depression, alcohol-related diseases, anxiolytics, hypnotics, sedatives, SSRIs, Charlson comorbidity index, education.  In addition adjusted for markers of smoking (inhaled corticosteroids, inhaled anticholinergics, diagnosis of COPD) | *(continued)*  **SABA**  >3 years vs. never use:  OR=0.76 (0.56-1.02)  **Salbutamol**  >3 years vs. never use:  OR=0.84 (0.56-1.26)  **Terbutaline**  >3 years vs. never use:  OR=0.70 (0.47-1.05)  **LABA**  >3 years vs. never use:  OR=0.57 (0.40-0.82)  **Salmeterol**  >3 years vs. never use:  OR=0.54 (0.34-0.86)  **Formoterol**  >3 years vs. never use:  OR=0.59 (0.35-1.00)  **With further adjustments for proxies of smoking**  **β2-agonists**  >3 years vs. never use:  OR=0.64 (0.42-0.98)  **Time lag**  “analysis applying different time lags (up to 5 years) did not lead to materially different estimates” |
| 10. | Koren et al., 2019  Israel | Cohort study  Maccabi Health Services  1998-2016 | Exposed cohort: 145,098 individuals on β-antagonists, 53.8 (19.5), 59.6%  Unexposed cohort:  1,187,151 individuals not on β-antagonists or β-agonists, 53.3 (19.5), 59.5% | β-antagonists | Ever vs. never  Different lags | Matched on age, sex, smoking status, LDL levels and statin use. | **β-antagonists**  1-year lag  Ever vs. never:  HR=1.51 (1.28-1.77)  2-year lag  Ever vs. never:  HR=1.70 (1.20-2.39)  4-year lag  Ever vs. never:  HR=1.86 (1.26-2.74)  6-year lag  Ever vs. never:  HR=1.84 (1.11-3.07)  8-year lag  Ever vs. never:  HR=1.70 (1.05-2.80) |
| 11. | Warda et al., 2019  Germany | Case-control study  Disease Analyzer database (IQVIA)  2013-2017 | Cases: 9,127 PD, 75.8 (9.6), 51.6%  Controls: 9,127 individuals without PD, 75.8 (9.6), 51.6% | β-antagonists ATC C07 | At-least-once vs. never  ≥3 years vs. <3 years  Effect per therapy year | Matched on age, sex, and treating physician  Age, sex, stroke, transient ischemic attack, depression, hypertension, antihypertensive drug prescriptions, ischemic heart diseases, heart rhythm disorders, heart failure, diabetes mellitus, renal failure. | **β-antagonists**  At-least-once vs. never:  OR=1.04 (0.97-1.11)  ≥3 years vs. <3 years:  OR=0.93 (0.87-1.00)  Effect per therapy year:  OR=1.00 (1.00-1.01) |
| 12. | de Germay et al.,  2020  France | Matched nested case-control study  EGB, ≥ 40 years old  2006-2017 | Cases: 2,225 incident PD cases, 75.6 (10.2), 51.0%  Controls: 2225 controls without PD, 75.6 (10.2), 51.0% | β-antagonists;  Propranolol;  β-agonists;  Salbutamol. | Ever vs. never  Different time lag  Duration of exposure | Matched on gender, birth year, and insurance scheme  Adjusted for arterial hypertension, calcium channel antagonists, antipsychotics, tobacco smoking, myocardial infarction, heart failure, dementia,  and migraine | **β-antagonists**  Time lag  1-year lag  Ever vs. never:  aOR=1.05 (0.91-1.20)  3-year lag  Ever vs. never:  aOR=0.98 (0.83-1.17)  Duration of exposure  <6 months vs. 0 months:  aOR=1.21 (0.99-1.49)  ≥6 months vs. 0 months:  aOR=0.95 (0.80-1.50)  **Propranolol**  Time lag  1-year lag  Ever vs. never:  aOR=2.11 (1.38-3.23)  3-year lag  Ever vs. never:  aOR=1.73 (1.07-2.81)  Duration of exposure  <6 months vs. 0 months:  OR=2.08 (1.28-3.39)  ≥6 months vs. 0 months:  OR=2.18 (0.91-5.52)  *(continues)* |
| 12. | De Germay et al.,  2020  France | Matched case-control study  EGB, ≥ 40 years old  2006-2017 | Cases: 2225 incident PD cases, 75.6 (10.2), 51.0%  Controls: 2225 controls without PD, 75.6 (10.2), 51.0% | β- antagonists;  Propranolol;  β-agonists;  Salbutamol. | Ever vs. never  Different time lag  Duration of exposure | Matched on gender, birth year, and insurance scheme  Adjusted for arterial hypertension, calcium channel antagonists, antipsychotics, tobacco smoking, myocardial infarction, heart failure, dementia,  and migraine | *(continued)*  **β-agonists, individuals with diabetes**  Time lag  1-year lag  Ever vs. never:  OR=1.61 (1.02-2.55)  3-year lag  Ever vs. never:  OR=1.03 (0.59-1.78)  Duration of exposure  <3 months vs. 0 months:  OR=2.66 (1.35-5.26)  ≥3 months vs. 0 months:  OR=1.06 (0.58-1.92)  **β-agonists, individuals without diabetes**  Time lag  1-year lag  Ever vs. never:  OR=0.75 (0.60-0.93)  3-year lag  Ever vs. never:  OR=0.96 (0.74-1.24)  Duration of exposure  <3 months vs. 0 months:  OR=0.60 (0.45-0.83)  ≥3 months vs. 0 months:  OR=0.89 (0.67-1.17)  *(continues)* |
| 12. | De Germay et al.,  2020  France | Matched case-control study  EGB, ≥ 40 years old  2006-2017 | Cases: 2225 incident PD cases, 75.6 (10.2), 51.0%  Controls: 2225 controls without PD, 75.6 (10.2), 51.0% | β-antagonists;  Propranolol;  β-agonists;  Salbutamol. | Ever vs. never  Different time lag  Duration of exposure | Matched on gender, birth year, and insurance scheme  Adjusted for arterial hypertension, calcium channel antagonists, antipsychotics, tobacco smoking, myocardial infarction, heart failure, dementia,  and migraine | *(continued)*  **Salbutamol, individuals with diabetes**  Time lag  1-year lag  Ever vs. never:  OR=1.85 (0.84-4.09)  3-year lag  Ever vs. never:  OR=1.73 (1.07-2.81)  Duration of exposure  <3 months vs. 0 months:  OR=2.27 (0.82-6.23)  ≥3 months vs. 0 months:  OR=1.09 (0.30-4.00)  **Salbutamol, individuals without diabetes**  Time lag  1-year lag  Ever vs. never:  OR=0.73 (0.52-1.01)  3-year lag  Ever vs. never:  OR=0.88 (0.60-1.29)  Duration of exposure  <3 months vs. 0 months:  OR=0.66 (0.44-0.98)  ≥3 months vs. 0 months:  OR=0.88 (0.48-1.62) |
| 13. | Chen et al., 2020  Canada | Matched nested case-control study  Cohort of 242,218 COPD patients  British Columbia health administrative databases  1997-2015 | Cases: 732 PD cases, 70.7 (8.6), 63%  Controls: 3660 controls without PD, 70.7 (8.6), 63% | β2-agonists | Regular use (≥ 1 dispensation every 6 months) vs. no use  Irregular use (≥1 dispensation overall, but with at least 6 months without prescriptions)  vs. no use | Matched on age, sex, calendar time, number of outpatient visits, number of dispensed medication for any cause (all within the 12months prior to COPD diagnosis).  Charlson index,  neighbourhood  household, COPD residence, # of hospital visits | **β2-agonists**  2-year lag, 4 years of follow up  Regular use vs. no use  HR=1.14 (0.93-1.40)  Irregular use vs. no use  HR=1.15 (0.92-1.45)  3-year lag, 5 years of follow up  Regular use vs. no use  HR=1.19 (0.95-1.49)  Irregular use vs. no use  HR=1.22 (0.95-1.58) |
| 14. | Giorgianni et al.,  2020  United Kingdom | Matched nested case-control study  CPRD, >50 years old  1995-2016 | Cases: 8,604 incident PD cases, 66.3 (9.9), 64.4%  Controls: 86,040 controls without PD, 66.3 (10.0), 64.4% | β2-agonists,  β-antagonists | Ever vs. never use  Different exposure time lags  Follow up time  Time since  1^st^ β2-agonist prescription  Cumulative duration of use  Cumulative duration of use and follow up  Cumulative duration of use and lag time | Matched on age, sex, date of cohort entry, and duration of follow up.  Further adjusted for BMI categories, alcohol abuse, smoking, comorbidities, use of other medications, # of visits to the doctor, use of β-antagonists and antimuscarinic drugs | **β2-agonists**  Any use vs. no use  HR=0.83 (0.75-0.91)  Time lag  None  Any use vs. no use  HR=0.84 (0.77-0.92)  2-year lag  Any use vs. no use  HR=0.85 (0.77-0.94)  3-year lag  Any use vs. no use  HR=0.87 (0.78-0.97)  5-year lag  Any use vs. no use  HR=0.97 (0.85-1.10)  Follow up time  1-5 years  Any use vs. no use  HR=0.66 (0.53-0.81)  7-13 years  Any use vs. no use  HR=0.90 (0.80-1.02)  14-22 years  Any use vs. no use,  HR=0.86 (0.70-1.06)  Time since 1^st^ β2-agonist prescription  1-2 years vs. no use  HR=0.75 (0.64-0.88)  3-5 years vs. no use  HR=0.81 (0.69-0.94)  6-21 years vs. no use  HR=0.91 (0.79-1.05)  *(continues)* |
| 14. | Giorgianni et al.,  2020  United Kingdom | Matched nested case-control study  CPRD, >50 years old  1995-2016 | Cases: 8,604 incident PD cases, 66.3 (9.9), 64.4%  Controls: 86,040 controls without PD, 66.3 (10.0), 64.4% | β2-agonists,  β-antagonists | Ever vs. never use  Different exposure time lags  Follow up time  Time since  1^st^ β2-agonist prescription  Cumulative duration of use  Cumulative duration of use and follow up  Cumulative duration of use and lag time | Matched on age, sex, date of cohort entry, and duration of follow up.  Further adjusted for BMI categories, alcohol abuse, smoking, comorbidities, use of other medications, # of visits to the doctor, use of β-antagonists and antimuscarinic drugs | *(continued)*  Cumulative duration of use, all follow up  <3 months vs. no use  HR=0.83 (0.72-0.94)  3-24 months vs. no use  HR=0.77 (0.66-0.88)  >24 months vs. no use  HR=0.97 (0.80-1.17)  Cumulative duration of use,  1-5 years of follow up  <3 months vs. no use  HR=0.63 (0.47-0.84)  3-24 months vs. no use  HR=0.66 (0.48-0.90)  >24 months vs. no use  HR=1.05 (0.53-2.08)  Cumulative duration of use,  7-13 years of follow up  <3 months vs. no use  HR=0.93 (0.78-1.10)  3-24 months vs. no use  HR=0.86 (0.71-1.04)  >24 months vs. no use  HR=0.93 (0.73-1.19)  Cumulative duration of use,  14-22 years of follow up  <3 months vs. no use  HR=0.86 (0.63-1.16)  3-24 months vs. no use  HR=0.69 (0.49-0.97)  >24 months vs. no use  HR=1.15 (0.81-1.62)  *(continues)* |
| 14. | Giorgianni et al.,  2020  United Kingdom | Matched nested case-control study  CPRD, >50 years old  1995-2016 | Cases: 8,604 incident PD cases, 66.3 (9.9), 64.4%  Controls: 86,040 controls without PD, 66.3 (10.0), 64.4% | β2-agonists,  β-antagonists | Ever vs. never use  Different exposure time lags  Follow up time  Time since  1^st^ β2-agonist prescription  Cumulative duration of use  Cumulative duration of use and follow up  Cumulative duration of use and lag time | Matched on age, sex, date of cohort entry, and duration of follow up.  Further adjusted for BMI categories, alcohol abuse, smoking, comorbidities, use of other medications, # of visits to the doctor, use of β-antagonists and antimuscarinic drugs | *(continued)*  Cumulative duration of use, no lag  <3 months vs. no use  HR=0.84 (0.74-0.96)  3-24 months vs. no use  HR=0.77 (0.67-0.89)  >24 months vs. no use  HR=1.01 (0.84-1.21)  Cumulative duration of use,  2-year lag  <3 months vs. no use  HR=0.88 (0.77-1.01)  3-24 months vs. no use  HR=0.75 (0.64-0.88)  >24 months vs. no use  HR=1.01 (0.82-1.24)  Cumulative duration of use,  3-year lag  <3 months vs. no use  HR=0.84 (0.72-0.98)  3-24 months vs. no use  HR=0.84 (0.71-0.99)  >24 months vs. no use  HR=1.04 (0.83-1.30)  Cumulative duration of use,  5-year lag  <3 months vs. no use  HR=0.96 (0.81-1.14)  3-24 months vs. no use  HR=0.96 (0.79-1.16)  >24 months vs. no use  HR=1.01 (0.75-1.35)  *(continues)* |
| 14. | Giorgianni et al.,  2020  United Kingdom | Matched nested case-control study  CPRD, >50 years old  1995-2016 | Cases: 8,604 incident PD cases, 66.3 (9.9), 64.4%  Controls: 86,040 controls without PD, 66.3 (10.0), 64.4% | β2-agonists,  β- antagonists | Ever vs. never use  Different exposure time lags  Follow up time  Time since  1^st^ β2-agonist prescription  Cumulative duration of use  Cumulative duration of use and follow up  Cumulative duration of use and lag time | Matched on age, sex, date of cohort entry, and duration of follow up.  Further adjusted for BMI categories, alcohol abuse, smoking, comorbidities, use of other medications, # of visits to the doctor, use of β-antagonists and antimuscarinic drugs | *(continued)*  **β-antagonists**  Any use vs. no use  HR=1.45 (1.37-1.54)  Cumulative duration of use, all follow up  <1 year vs. no use  HR=1.70 (1.57-1.85)  1-5 years vs. no use  HR=1.39 (1.28-1.52)  >5 years vs. no use  HR=1.13 (1.00-1.27) |
| 15. | Marras et al., 2020  Canada | Matched nested case-control study  Ontario’s health databases, patients >65 years with COPD/asthma  1997-2017 | Cases: 3,568 incident PD cases, 79.40 (6.14), 61.8%  Controls: 17,822 controls**,** 79.43 (6.15), 61.8% | LABA,  SABA. | Duration of exposure to the drug | Matched on sex, date of birth, cohort entry date, and condition (asthma/COPD)  Further adjusted for dementia at study entry, Charlson comorbidity index, medications dispensed in the year prior to cohort entry, and number of inpatients stays in the year preceding cohort entry. | **SABA**  HR=0.90 (0.86-0.96) “per additional month of exposure”  **HR for disease progression (from PD diagnosis to dementia, results not included in meta-analysis)**  **LABA**  Any use vs. no use  HR=1.09 (0.85-1.40)  **SABA**  Any use vs. no use  HR=1.22 (0.92-1.62) |
| 16. | Paakinaho et al.,  2022  Finland | Matched nested  case-control study  PD Register (FINPARK) linked to prescription database  1996-2015 | Cases: 1,406 clinically verified PD cases with asthma/COPD diagnosed at least 3 years prior to PD, 72.7 (8.8), 51.1%  Controls: 8,630 with asthma/COPD and no PD, 72.9 (8.2), 50.6% | Any β2-agonists;  LABA (salmeterol, formoterol, indacaterol, olodaterol, and vilanterol);  SABA (salbutamol, terbutaline, fenoterol).  Exposure only up to 3 years prior to index date | Quartiles of defined daily doses (DDDs) among all users | Matched on age, sex, duration of asthma/COPD, time since pulmonary diagnosis, and region.  Further adjusted for cancer history, cardiovascular diseases, diabetes, socioeconomic status, stroke, substance abuse and traumatic brain injury. | **Any β2-agonists (n = 7851)**  **Cumulative DDD**  150-555 DDD vs. 6-148 DDD:  aOR=1.11 (0.93-1.32)  560-1848 DDD vs. 6-148 DDD:  aOR=1.06 (0.89-1.27)  1850-15246 DDD vs. 6-148 DDD:  aOR=0.85 (0.70-1.03)  **SABA (n = 7376)**  **Cumulative DDD**  78-223 DDD vs. 7-75 DDD:  aOR=1.06 (0.89-1.27)  225-633 DDD vs. 7-75 DDD:  aOR=0.98 (0.82-1.17)  635-14016 DDD vs. 7-75 DDD:  aOR=0.84 (0.69-1.02)  **LABA (n = 7851)**  **Cumulative DDD**  225-900 DDD vs. 15-240 DDD:  aOR=0.94 (0.73-1.21)  915-2100 DDD vs. 15-240 DDD:  aOR=0.89 (0.69-1.16)  2115-11010 DDD vs. 15-240 DDD:  aOR=0.88 (0.67-1.16)  *(continues)* |
| 16. | Paakinaho et al.,  2022  Finland | Matched nested  case-control study  PD Register (FINPARK) linked to prescription database  1999-2015 | Cases: 1,406 clinically verified PD cases with asthma/COPD diagnosed at least 3 years prior to PD, 72.7 (8.8), 51.1%  Controls: 8,630 with asthma/COPD and no PD, 72.9 (8.2), 50.6% | Any β2-agonists,  LABA (salmeterol, formoterol, indacaterol, olodaterol, and vilanterol),  SABA (salbutamol, terbutaline, fenoterol).  Exposure only up to 3 years prior to index date | Quartiles of defined daily doses (DDDs) among all users  Quartiles of annual exposure | Matched on age, sex, duration of asthma/COPD, time since pulmonary diagnosis, and region.  Further adjusted for cancer history, cardiovascular diseases, diabetes, socioeconomic status, stroke, substance abuse and traumatic brain injury. | *(continued)*  **Any β2-agonists**  **Annual exposure**  50-120 vs. 6-49 DDD/year:  aOR=1.16 (0.97-1.38)  121-259 vs. 6-49 DDD/year:  aOR=1.11 (0.92-1.33)  260-1691 vs. 6-49 DDD/year:  aOR=0.92 (0.76-1.11)  **SABA**  **Annual exposure**  34-57 vs. 7-33 DDD/year:  aOR=1.01 (0.85-1.20)  58-113 DDD/year vs. 7-33 DDD/year:  aOR=0.93 (0.78-1.12)  114-1533 vs. 7-33 DDD/year:  aOR=0.91 (0.75-1.10)  **LABA**  **Annual exposure**  107-210 vs. 15-105 DDD/year:  aOR=0.91 (0.71-1.17)  212-310 vs. 15-105 DDD/year:  aOR=0.94 (0.73-1.21)  311-1032 vs. 15-105 DDD/year:  aOR=0.75 (0.58-0.97)  *(continues)* |
| 16. | Paakinaho et al.,  2022  Finland | Matched nested  case-control study  PD Register (FINPARK) linked to prescription database  1996-2015 | Cases: 1,406 clinically verified PD cases with asthma/COPD diagnosed at least 3 years prior to PD, 72.7 (8.8), 51.1%  Controls: 8,630 with asthma/COPD and no PD, 72.9 (8.2), 50.6% | Any β2-agonists,  LABA (salmeterol, formoterol, indacaterol, olodaterol, and vilanterol),  SABA (salbutamol, terbutaline, fenoterol).  Exposure only up to 3 years prior to index date | Quartiles of defined daily doses (DDDs) among all users  Quartiles of annual exposure | Matched on age, sex, duration of asthma/COPD, time since pulmonary diagnosis, and region.  Further adjusted for cancer history, cardiovascular diseases, diabetes, socioeconomic status, stroke, substance abuse and traumatic brain injury. | *(continued)*  **β-agonists, individuals with both COPD and asthma**  **Quartiles of annual exposure**  2^nd^ vs. 1^st^ quartile  aOR=0.65 (0.36-1.19)  3^rd^ vs. 1^st^ quartile  aOR=0.48 (0.27-0.87)  4^th^ vs. 1^st^ quartile  aOR=0.68 (0.38-1.20)  **β-agonists, individuals with COPD only**  **Quartiles of annual exposure**  2^nd^ vs. 1^st^ quartile  aOR=0.77 (0.21-2.76)  3^rd^ vs. 1^st^ quartile  aOR=1.96 (0.51-7.46)  4^th^ vs. 1^st^ quartile  aOR=1.13 (0.29-4.32)  **β-agonists, individuals with asthma only**  **Quartiles of annual exposure**  2^nd^ vs. 1^st^ quartile  aOR=1.02 (0.77-1.36)  3^rd^ vs. 1^st^ quartile  aOR=1.11 (0.83-1.49)  4^th^ vs. 1^st^ quartile  aOR=0.74 (0.55-1.01) |
| 17. | Nadeem et al., 2021  United States | Cohort study  Random sample of 20% of Medicare beneficiaries, >65 years with asthma, COPD, and/or bronchiectasis  2007–2014 | Cohort: 236,201 individuals  Exposed to β2-agonist: 53.7% (126,882)  Mean age 77 years old, 32% | β2-agonists | Exposed vs. unexposed to β2-agonist | Adjusted for smoking history, severity of asthma and COPD, and use of other drugs,  sex, race or ethnicity, Low Income Subsidy status, disease status/ | **β2-agonists**  # of 30-day claims  Exposed vs. Unexposed  OR=1.656 (1.636-1.676)  # of claims, interacted with time (months)  Exposed vs. Unexposed  OR=0.986 (0.977-0.995) |
| 18. | Tuominen et al.,  2023  Norway | Population-based cohort  PD Registry linked to the national prescription database  2005–2019 | Exposed to  β2-agonists: 251,302  SABA: 418,506  LABA: 360,467  Ultra-LABA: 68,953  Unexposed: 2,600,086 individuals | β2-agonists  SABA  LABA  Ultra-LABA | Exposed (≥2 prescriptions) vs. unexposed  (<2 prescriptions) | Adjusted for age, education, sex, comorbidity. | **Β2-agonists**  Exposed vs. unexposed  HR=0.84 (0.80-0.88)  **SABA**  Exposed vs. unexposed  HR=0.84 (0.79-0.89)  **LABA**  Exposed vs. unexposed  HR=0.85 (0.81-0.90)  **Ultra-LABA**  Exposed vs. unexposed  HR=0.60 (0.49-0.73)  *(continues)* |
| 18. | Tuominen et al.,  2023  Norway | Population-based cohort  PD Registry linked to the national prescription database  2005–2019 | Exposed to  β2-agonists: 251,302  SABA: 418,506  LABA: 360,467  Ultra-LABA: 68,953  Unexposed: 2,600,086 individuals | β2-agonists  SABA  LABA  Ultra-LABA | Exposed (≥2 prescriptions) vs. unexposed  (<2 prescriptions) | Adjusted for education, sex, comorbidity. | *(continued)*  Patients with history of COPD excluded (further accounts for smoking)  **Β2-agonists**  Exposed vs. unexposed  HR=0.85 (0.76-0.94)  **SABA**  Exposed vs. unexposed  HR=0.91 (0.0.83-1.00)  **LABA**  Exposed vs. unexposed  HR=0.92 (0.80-1.05)  **Ultra-LABA**  Exposed vs. unexposed  HR=0.72 (0.44-1.18) |
| 19. | Liu et al.,  2023  Sweden | Cohort  Longitudinal Integration Database for Health Insurance and Labor Market Studies  2005-2013 | Cohort: 5,186,886 individuals  Exposed to β2-agonist: 430,885  906 primary diagnoses of PD identified  74.7 (10.1 years), 49.2% | β2-agonists  SABA  LABA | Exposed vs. unexposed to β2-agonist | Adjusted to all anti-asthmatic drugs, age, sex, birth year, country of birth, education, comorbidity index, COPD. | **β2AR agonists overall**  Exposed vs. unexposed:  HR=0.98 (0.87–1.09)  **β2AR agonists, short acting**  Exposed vs. unexposed:  HR=1.01 (0.91–1.12)  **β2AR agonists, short acting, low dose**  Exposed vs. unexposed:  HR=0.97 (0.85–1.11)  **β2AR agonists, short acting, high dose**  Exposed vs. unexposed:  HR=1.04 (0.91–1.19)  **β2AR agonists, long acting**  Exposed vs. unexposed:  HR=0.96 (0.84–1.09)  **β2AR agonists, long acting, low dose**  Exposed vs. unexposed:  HR=0.91 (0.77–1.08)  **β2AR agonists, long acting, high dose**  Exposed vs. unexposed:  HR=0.99 (0.84–1.18) |
| 20. | Feng et al.,  2023  United Kingdom | Cohort  UK Biobank  Total of 1,297,332 person-years, median of 12.8 years of follow-up | Cohort: 105,763 participants  Mean 60.7 (6.48), 46.3  1130 cases of PD in participants | β-antagonists  β-antagonists, non-selective | Users vs. non-users | Adjusted by age, sex, ethnicity, smoking status, alcohol use, body mass index, anxiety, socioeconomic status, deprivation score, income level, educational level, hypertension, the duration of hypertension, diabetes, hyperlipidaemia, cerebrovascular diseases and the PD polygenic risk score. | **β-antagonists**  Any use vs. no use:  HR=0.99 (0.85-1.15)  **β-antagonists, non-selective**  Any use vs. no use:  HR=1.47 (1.04-2.06)  **β-antagonists, non-selective, excluding participants who developed PD outcomes within the first year of follow-up**  Any use vs. no use:  HR=1.42 (1.00-2.00)  **β-antagonists, non-selective,**  **excluding tremor**  Any use vs. no use:  HR=1.51 (1.08-2.12)  **β-antagonists, non-selective,**  **adjusting number of long-term conditions**  Any use vs. no use:  HR=1.41 (1.01-1.98)  **β-antagonists, non-selective,**  **from competing risk models**  Any use vs. no use:  HR=1.53 (1.02-2.29) |
| 21. | Jung et al., 2023  South Korea | Cohort  National Health Insurance Service of Korea  Exposure period from 2003 to 2007; observation period 2008 to 2013 | Cohort of 1,025,340 participants  Adults > 60 years of age  Mean age PD group 71 years, non-PD 68.4 years | β2-agonists | Ever vs. never use | No | **β2-agonists**  Users vs. never users:  HR=1.51 (1.03-2.21) |
| 22. | Wei et al., 2023  Taiwan | Cohort  Taiwan's National Health Insurance Research Database  2008-2015 | Control cohort of 80,741 no medication  NP, NP | β-antagonists  β2-agonists  Betaxolol | Users vs. non-users | Matched on age and sex | **β-antagonists**  Users vs. non-users:  HR= 1.93 (1.74-2.15)  **Betaxolol**  Users vs. non-users:  HR=1.41 (0.9-2.21)  **β2-agonists**  Users vs. non-users:  HR=0.98 (0.87-1.11)  “Acebutolol, Atenolol, Bisoprolol, Metoprolol, Alprenolol, Carteolol, Carvedilol, Labetalol, Pindolol, Propranolol and Timolol, were found significant increased risk for PD”  “Fenoterol, Formoterol, Procaterol, Salbutamol, Salmeterol, Terbutaline and Trimetoquinol did not show reduced aHR for PD” |

**Abbreviations:** EGB = Echantillon Généraliste des Bénéficiaires; CHS: Clalit Health Services; CCAE = IBM MarketScan® Commercial Claims and Encounters database; CPRD = Clinical Practice Research Datalink; FINPARK = Finnish Parkinson's Disease registry cohort; GHC = Group Health Cooperative; GPRD = General Practice Research Database; MDCR = IBM MarketScan® Medicare Supplemental Database; MDCR = IBM MarketScan® Multi-State Medicaid Database; NorPD = Norwegian Prescription Database; Optum = Optum: De-Identified Clinformatics® Data Mart Database; NP =not provided.

## **Appendix 4.** Forest plot of the studies that includes selected populations of patients with respiratory conditions (chronic obstructive pulmonary disease, asthma and/or bronchiectasis).
